# A New Mother-Child Play Activity Program to Decrease Parenting Stress and Improve Child Cognitive Abilities: A Cluster Randomized Controlled Trial

**DOI:** 10.1371/journal.pone.0038238

**Published:** 2012-07-27

**Authors:** Yoshiyuki Tachibana, Ai Fukushima, Hitomi Saito, Satoshi Yoneyama, Kazuo Ushida, Susumu Yoneyama, Ryuta Kawashima

**Affiliations:** 1 Division of Maternal-Child Psychiatry, Department of Psychosocial Medicine, National Center for Child Health and Development, Tokyo, Japan; 2 Department of Applied Brain Science, Smart Aging International Research Center, IDAC, Tohoku University, Sendai, Japan; 3 Department of Functional Brain Imaging, IDAC, Tohoku University, Sendai, Japan; 4 Wakakusa Kindergarten, Sendai, Japan; The University of Queensland, Australia

## Abstract

**Background:**

We propose a new play activity intervention program for mothers and children. Our interdisciplinary program integrates four fields of child-related sciences: neuroscience, preschool pedagogy, developmental psychology, and child and maternal psychiatry. To determine the effect of this intervention on child and mother psychosocial problems related to parenting stress and on the children's cognitive abilities, we performed a cluster randomized controlled trial.

**Methodology/Principal Findings:**

Participants were 238 pairs of mothers and typically developing preschool children (ages 4–6 years old) from Wakakusa kindergarten in Japan. The pairs were asked to play at home for about 10 min a day, 5 days a week for 3 months. Participants were randomly assigned to the intervention or control group by class unit. The Parenting Stress Index (PSI) (for mothers), the Goodenough Draw-a-Man intelligence test (DAM), and the new S-S intelligence test (NS-SIT) (for children) were administered prior to and 3 months after the intervention period. Pre–post changes in test scores were compared between the groups using a linear mixed-effects model analysis. The primary outcomes were the Total score on the child domain of the PSI (for child psychosocial problems related to parenting stress), Total score on the parent domain of the PSI (for maternal psychosocial problems related to parenting stress), and the score on the DAM (for child cognitive abilities). The results of the PSI suggested that the program may reduce parenting stress. The results of the cognitive tests suggested that the program may improve the children's fluid intelligence, working memory, and processing speed.

**Conclusions/Significance:**

Our intervention program may ameliorate the children's psychosocial problems related to parenting stress and increase their cognitive abilities.

**Trial Registration:**

UMIN Clinical Trials Registry UMIN000002265

## Introduction

Vygotsky regarded play as the supreme educative activity of the preschool years; play contains all developmental tendencies in a condensed form and play is, itself, a major source of development [1]. On the other hand, it is suggested that encouraging parents and children to play together may serve as a vehicle to improve relationships in the family system [2]. Based on these ideas, we propose a new play activity intervention program for mother and child. This program was designed integrating various interdisciplinary areas for children, namely brain science, preschool pedagogy, developmental psychology, and child and maternal psychiatry. The purpose of the present study was to identify the effects of this intervention program on mother and child psychosocial problems related to parenting stress and on the children's cognitive abilities. We designed the program with consideration of the four points mentioned below.

First, the play activities in the program were designed based on the standard cognitive tasks associated with prefrontal cortex (PFC) functions. This design was chosen because PFC functions are deeply associated with children's social and emotional development [3]. What is more, PFC plays major roles in higher cognitive functions necessary for maintaining a healthy social life [4]. We targeted preschool children because executive function (EF), which is a major function of PFC, develops throughout the preschool period [5]. During the preschool years, enhancement of EF is regarded as one of the most important factors for healthy development [6]. On the other hand, Thorell and colleagues indicated that training PFC functions, such as working memory (WM), significantly improves not only the trained abilities but also the non-trained abilities in, for example, the spatial and verbal domains [7]. This indicates that training for PFC function has transfer effects to other non-trained abilities. Therefore, we associated the play activities with PFC functions in the design. To enhance child cognitive abilities in our play program, the play activities were designed based on standard cognitive tasks, such as WM, which were confirmed by previous neuroimaging studies to be associated with PFC [8], [9].

Second, we based the program design on the “zone of proximal development (ZPD)” and “scaffolding”, both concepts from preschool pedagogy and developmental psychology. Vygotsky defined the ZPD as “the distance between the actual developmental level as determined by independent problem solving, and the level of potential development as determined through problem solving under adult guidance or in collaboration with more capable peers” [1]. According to Vygotsky, the role of education is to provide children with experiences that are in their ZPDs-activities that challenge children but that can be accomplished with sensitive adult guidance. Vygotsky suggested that play creates a ZPD in children. For this reason, we created ZPDs for the children in our parent-child play program. We also encouraged “scaffolding” which is a metaphor for effective teaching/learning interactions within the ZPD (imagine the scaffold of a building under construction). This is because effective parent-child interaction within the ZPD fosters general cognitive growth and increases children's cognitive and social development [10]. Based on this idea, we designed the play activities within children's ZPDs as much as possible.

Third, we focused on the mother's emotional sensitivity to her child during the play activities. Our program was aimed at increasing positive mother–child interactions and reducing child and maternal psychosocial problems related to parenting stress. Parenting stress is not unusual; most parents experience it at some point [11]. However, it is an important risk factor for the development of dysfunctional parenting behaviors or behavior problems in the child [12]. Parenting stress, the most significant factor associated with hindering the development of social competence in children [13], is thought to be caused by child and maternal psychosocial problems [14]. Thus, an intervention that improves the psychosocial problems associated with parenting stress would benefit child development. Promoting positive mother–child interactions has been shown to improve child and mother psychosocial problems related to parenting stress [15]. Meta-analyses assessing the effectiveness of various types of attachment-based interventions have revealed that interventions focusing on maternal emotional sensitivity to children were the most effective in creating a positive attachment between the mother and child [16]. The concept of maternal emotional sensitivity was initially used in relation to mothers' caring for infants during their first year of life [17]. However, studies of interventions for toddlers and their mothers revealed that encouraging maternal emotional sensitivity improved mother–child interactions [15], [18], [19]. For that reason, our program instructed mothers to praise and show acceptance of their children as a means of improving maternal sensitivity and the child–mother relationship as one way to lower parenting stress.

Fourth, we made the program as undemanding on participants as possible. Bakermans-Kranenburg and colleagues [16] suggested that the lower the load of the intervention is on the participants, the more effective it is likely to be.

We hypothesized that our program would improve child and maternal psychosocial problems related to parenting stress. Furthermore, we hypothesized that our play activities would increase the child's cognitive abilities. To test these hypotheses, we conducted a cluster randomized controlled trial.

## Methods

### Ethics statement

The study protocol was reviewed and approved by the Institutional Review Board of the Tohoku University Graduate School of Medicine. Based on the declaration of Helsinki, written informed consent was taken from each family.

### Subjects

We recruited pairs of mothers and their children from a private kindergarten in Sendai, Japan. The inclusion criterion of this study was children aged 4 to 6 in the kindergarten and their mothers who consented on participating in this study. Two hundred and thirty eight pairs of mothers and children participated in this study. The children included of 115 boys and 123 girls, their ages ranging from four to six years old. The mean age of the children was 5.13 (SD = 0.586). The mean age of the parents was 33.78 (SD = 4.12). Fourteen children (8 children in the intervention group and 6 children in the control group) were raised in families where the father was absent. Two hundred and twenty four children were raised in dual-parent families. The exclusion criterion of this study was a current clinically severe illness or disorder which made it impossible to perform the intervention program. Additionally, no children in this study had histories of developmental disorders or psychiatric diseases.

**Figure 1:**
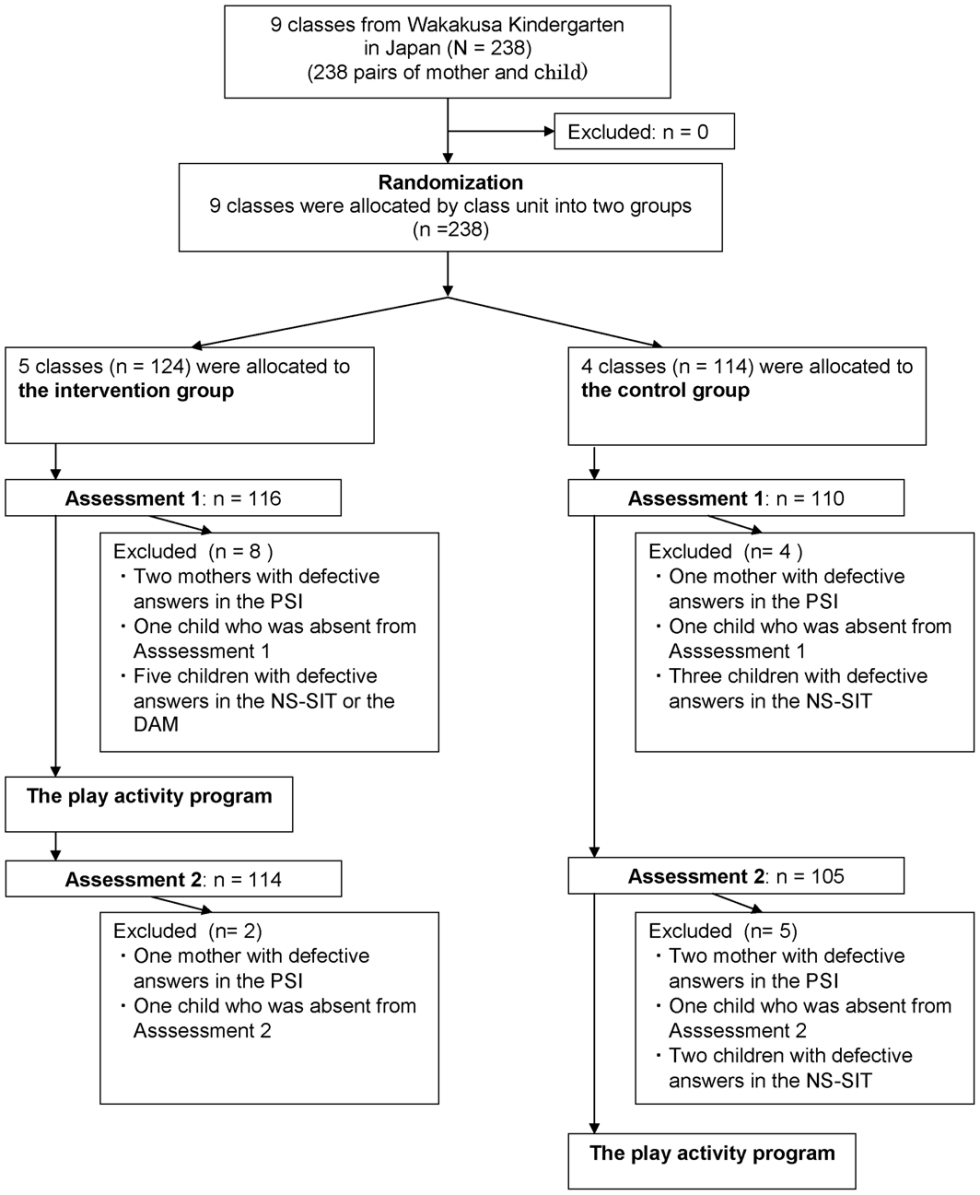
Trial profile.

We ran the trial in accordance with the CONSORT statement [20] (Figure 1). The protocol for this trial and supporting CONSORT checklist are available as supporting information; see Checklist S1 and Protocol S1. To ensure comparability of the intervention and comparison groups with respect to baseline participants' psychosocial problems related to parenting stress and child cognitive abilities, stratified randomization was performed according to grade level. After consent was obtained, a randomization manager who did not know anything about either the participants or the classes assigned the classes with sequential identification numbers. The numbers were randomly allocated to the intervention group or the control group with concealment based on the opaque sealed envelope randomization method. The classes' teachers were given the sealed numbers by a kindergarten staff member who was not involved in this study, and the classes were allocated into the two groups. Additionally, the allocation was kept separate from the executor of the number assignment. The assessors of the tests and the analyzer of the data were unaware of the allocation; however, the allocation could not be masked from the participants. Strict separation was kept between assessment and data analysis; assessors and data analyzers were located separately.

The sample size of the present study was predefined as nearly the number of children in the kindergarten. The study was performed as part of the kindergarten curriculum, and we recruited children in the middle and the upper levels of Wakakusa Kindergarten.

Both groups participated in the same kindergarten curriculum and continued their usual daily lives at home. Only the participants of the intervention group were asked to participate in the play program for mother and child at home five days a week for the first three months. To measure the efficacy of our program, after pre-admission testing, there were two major assessment sessions identified as Assessment 1 and Assessment 2. The test battery, consisting of experimental cognitive tests for the children and questionnaires about parenting stress for the parents, was administered before (Assessment 1) and after (Assessment 2) the intervention group underwent the intervention program. Assessment 1 was conducted to confirm whether the two groups were equivalent before they participated in the program. We assessed the effectiveness of the program with the Assessment 1 – Assessment 2 changes between the two groups by the two sample *t*-tests. We used a waiting-list control group design. After Assessment 2, the control group took the intervention program. This was done so that both of the groups could experience the program.

### The play intervention program (Appendix S1)

The play program was made by the teachers of the kindergarten and the neuroscientists in our laboratory. The program consists of a set of play activities based on standard cognitive tasks used in previous neuroimaging studies. We intended to design the play activities to be associated with PFC as shown in the results of previous imaging studies. The contents of the play activities were updated monthly (four play activities per month).

We made the program with consideration for the enhancing factors of scaffolding. Research showed that effective scaffolding has the following components and goals [21]: 1) Joint problem solving; what is important is that children interact with someone while the two are jointly trying to reach a goal. 2) Intersubjectivity; this is the process whereby two participants who begin a task with different understandings arrive at a shared understanding. 3) Warmth and responsiveness; Children's engagement with a task and willingness to challenge themselves are maximized when collaboration with the adult is pleasant, warm, and responsive and the adult gives verbal praise and attributes competence to the child, as appropriate. 4) Keeping the child in the ZPD; this is usually achieved in two ways; I) structuring the task and the surrounding environment so that the demands on the child at any given time are at an appropriately challenging level, and II) constantly adjusting the amount of adult intervention to the child's current needs and abilities. 5) Promoting self-regulation; a combination of active withdrawal by the adult in response to active takeover by the child is crucial for the development of self-regulation. We made our program based on these five factors. For joint problem solving, the mothers and children developed mutual objectives through the play activities in our program. The mothers and children were asked to alternate roles in the play activities in order to foster intersubjectivity. To encourage warmth and responsiveness, the mothers were asked to be pleasant, warm, and responsive during the play activities. To keep the children in the ZPD, the difficulty levels of the play activities could be adjusted according to the children's ability levels. To promote self-regulation, we asked mothers to be responsive to the children's contributions and to build on them according to children's initiative in the play activities.

In order to become familiar with the activities, the children did the play activities of the program with their teachers and their classmates at the kindergarten before they played at home. After that, children were asked to take the initiative and teach their mothers how to do the play activities. Mothers were asked to play together with the children according to the children's initiatives. This program comprised four play activities each month. Those four play activities were selected so as to have different targets of the cognitive ability. Hand-outs of the play activities were also distributed to the mothers so they could perform the program correctly. In the hand-outs, the four play activities corresponded to four different colors; red, yellow, green, blue. The participants were given a calendar and 20 seals (i.e. 4 colors×5 times per one play activity) for each month. The children were asked to put the corresponding seal of a play activity on the calendar after they performed the activity. Every day the children were free to select the play activities they wanted to perform most out of the four. This calendar-seal system made it possible for the participants to perform the four play activities evenly every month. In addition, this system was performed to improve the participants' commitment to the program. However, we did not check the participants' calendars after this program.

We made the load of this program as easy as possible on the participants since the meta-analysis of intervention studies for enhancing mother-child attachment revealed a lesser intervention load to be most effective [16]. The play activities took approximately ten minutes to perform each day. This time period was based on that used in the previous studies [22], [23], and the kindergarten teachers agreed that 10 min was an appropriate length of time for the children to concentrate on play activities and enjoy them. Thus, we regarded a length of “ten minutes” to be the most suitable length for a play session for preschool children and their mothers for this intervention program. The entire period of this program was set at three months, because the period of most effective interventions is from one to six months [16]. If the number of intervention sessions is too large, the participants may get bored and not be able to enjoy the program. On the other hand, if the number of intervention sessions is too small, the full effect of the program cannot be observed. We regarded the period of “three months” to be appropriate for this program. In addition, we set the frequency of the program to five times a week because many effective cognitive interventions studies in the past performed their programs five times a week [7], [24]. This frequency also meant that the participants could take a break from the program two days a week, which was thought to be effective for improving participants' compliance with the program. Thus, we asked participants to perform the program ten minutes a day, five times a week, for three months. The meta-analysis of attachment-based interventions also indicated that use of non-professional interveners is more effective than that of professional interveners, and that interventions with no-personal contact, such as those using video tapes, are also quite effective [16]. Therefore, we did not use professional interveners and simply asked the mothers to enjoy the program with their children, taking care to express maternal emotional sensitivity while they played. We asked the mothers to follow the intervention program described in the orientation session held before the intervention period.

### Assessment tests

We assessed child and mother psychosocial problems related to parenting stress and child cognitive abilities using the tests mentioned below.

#### 1) The parenting stress index (PSI)

The PSI was used to assess child and maternal psychosocial problems related to parenting stress. The PSI is a self-reported questionnaire by parents and has been standardized with parents and children ranging in age from 1 month to 12 years in different cultures and is considered to be a reliable instrument across cultures [25], [26]. It has been standardized in Japan [27]. The PSI consists of a child domain and a parent domain. The child domain score reflects parental stress associated with the child's individual characteristics as measured on 6 subscales: Distractibility/Hyperactivity, Adaptability, Reinforces parent, Demandingness, Mood, and Acceptability. The parent domain reflects parental stress associated with the parental role and is measured on 7 subscales: Competence, Isolation, Attachment, Health, Role restriction, Depression, and Spouse. “Total Stress” score is obtained by adding the scores of the child domain and parent domain subscales. Higher scores indicate more stress. Decreases in the PSI scores indicate favorable outcomes associated with the intervention. In this study, the mothers were asked to fill out the PSI.

#### 2) The Goodenough draw-a-man intelligence test (DAM)

The DAM is an international standard cognitive test for assessing child cognitive abilities. Children are instructed to draw a picture of man freely on a paper. Children's intelligence quotients are objectively assessed on the pictures they draw by the scoring system. This test was developed by Florence Goodenough under the assumption that individual differences in intellectual ability are reflected in the details included in a drawing of a human figure [28]. Its validity and reliability has been indicated in many countries which have wide varieties of social and cultural backgrounds [29]. It has also been standardized in Japan [30]. Increased IQ scores in the DAM indicate that children improved their performance on this test. Increases in DAM scores reflect favorable outcomes associated with the intervention. Regarding assessment of the DAM, three graduate students whose majors were educational psychology and who were accustomed to assessing cognitive tests for children were trained for 6 hours before Assessment 1. After the training, we checked their assessment. The reliability of their assessments could be regarded as equivalent. The three assessors assessed the DAM for Assessment 1 and Assessment 2.

#### 3) The new S-S intelligence test (NS-SIT)

Vygotsky regarded cognition as a social phenomena [1]. Therefore, we assessed child cognitive abilities from the view of social context. The NS-SIT was developed and standardized by K. Yamane [31]. It assesses preschool child cognitive abilities through tasks based on social context and daily life situations in Japanese culture. Its subscales consist of “Understanding relationships between things”, “Counting and comparing numbers”, “Calculation”, “Completion of pictures”, “Working memory”, and “Processing speed”. Each section has an exercise before the assessment test to make children fully understand how to perform it. Increases in the NS-SIT scores indicate improved cognitive abilities and favorable outcomes associated with the intervention. “Understanding relationships between things” assesses the ability to infer how things are related. One question has a marked picture and between four and six other pictures in a box. Children are instructed to select the picture among pictures in the box which is related to the marked picture. Children answer ten questions within one minute in this section. In “Counting and comparing numbers”, children are asked to count the number of pictures depicted in three boxes. They are then asked to select the box in which the number of pictures is the second largest among the three (e.g. the numbers of ants depicted in the three boxes are three, five, and seven, respectively. The answer is the box in which 5 ants are depicted.). In “Counting”, one question has two boxes in which cubes are depicted. Children are instructed to count the number of cubes depicted in the two boxes and draw dots equal to the sum of the numbers of cubes. (e.g. three and two cubes are shown in the two boxes, respectively. Children should draw five dots.) There are ten questions in this section and children must answer them within one minute. In each question in “Completion of pictures”, a marked picture is divided into four parts, and one part is missing. Children are instructed to select the picture which corresponds to the missing part among the four pictures in the box. This section resembles Raven Colored Matrices [32]. There are ten questions in this section, and children must answer them in one minute. In “Working memory”, two stories are read by an examiner. With regard to each story, children are asked to select the pictures which best fit the examiner's descriptions. (e.g. the examiner reads a children's story, “There was a visitor at home. Mom served cookies and tea for him. It was hot. So mom brought an electric fan for him.” The examiner then asks, “What did mom bring to the visitors? Select from among the pictures.” The children then select three pictures among seven which correspond to what the Mom brought the visitor.) Children answer each question within one minute. “Processing speed” resembles the “coding and symbol search” in Wechsler Intelligence Scale for Children – III [33]. Children are instructed to draw lines corresponding to pictures. The numbers of lines which children should draw are different depending on pictures (i.e. car: one, bicycle: two, airplane: three lines, respectively). There are twenty-one pictures and children answer them within one minute.

### Outcomes

#### 1) Child and mother psychosocial problems related to parenting stress

The primary outcomes were “the Total score of the child domain” (for child psychosocial problems related to parenting stress) and “the Total score of the parent domain” of the PSI (for maternal psychosocial problems related to parenting stress). The secondary outcomes for child psychosocial problems related to parenting stress were the subscales of the child domain; Distractibility/Hyperactivity, Adaptability, Reinforces parent, Demandingness, Mood, Acceptability. The secondary outcomes for maternal psychosocial problems related to parenting stress were the subscales of the parent domain; Role restriction, Isolation, Spouse Competence, Depression, Attachment, Health).

#### 2) Child cognitive abilities

The primary outcome was Intelligence Quotient (IQ) in the DAM. The secondary outcomes were the subscales of the new S-S Intelligence Test (NS-SIT); “Understanding relationships”, “Understanding relationships between things”, “Counting and comparing numbers”, “Calculation”, “Completion of pictures”, “Working memory”, and “Processing speed”.

### Assessment of the effectiveness of the intervention

At the kindergarten, we performed Assessment 1 and Assessment 2 for the children using a single-blind method. The subjects were assigned to 2 groups masked to the examiner of the assessments and to those who inputted the data and who rated the tests. During Assessment 1, two mothers from the intervention group and one mother from the control group who skipped some questions in the PSI were excluded from the analysis. One child from the intervention group was excluded for being absent from the assessment. Five children from the intervention group with defective answers in either the NS-SIT or the DAM were excluded (they went to the toilets or quitted to answer during the test). Three children from the control group with defective answers in the NS-SIT were also excluded (they also went to the toilets or quitted to answer during the test). During Assessment 2, one mother from the intervention group and two mothers from the control group who skipped some questions in the PSI were excluded from the analysis. One child in both the intervention group and the control group was absent from Assessment 2. Two children who went to the toilets during the test in the NS-SIT were excluded. One hundred and fourteen mother-child pairs from the intervention group and one hundred and five pairs from the control group were analyzed for the assessments.

### Fidelity assessment

We performed the fidelity assessment of this program for the intervention group in Assessment 2. We asked them, “How often do you perform this program?”. The participants selected one among the following alternatives, “1. Every day, 2. About once every two days, 3. About twice a week, 4. About once a week, 5. Less than once every ten days”.

### Statistical analyses

To assess whether there were any significant differences in the children's and mothers' ages between the intervention group and the control group, two sample *t*-tests were performed. To assess whether there were any significant differences in the scores of Assessment 1 between the intervention group and the control group, two sample *t*-tests were performed. Changes in child and mother psychosocial problems related to parenting stress and in the children's cognitive abilities were assessed using linear mixed-effects models. The model analyses were constructed using the pre–post changes (i.e. the changes between Assessment 1 and Assessment 2) as dependent variables, group (intervention group and control group) as the fixed effect, and the kindergarten classes as the random effect. Statistical significance was set at *p*≤0.05. The effect size for between-group subscale changes was calculated using Cohen's d statistic [34]. Within-class variances in the outcome measures were calculated as the product of the diagonal compound symmetry correlation (CSR) and the intraclass correlation in the estimation of covariance parameters in the linear models [35], [36], [37]. The between-class variances in the outcome measures were calculated by subtracting the diagonal CSR from the within-class variances in the outcome measures [35], [36], [37]. All statistical analyses were carried out using the Statistical Package for the Social Sciences 16.0 (SPSS, Chicago, IL, USA).

## Results

Participants were recruited in July 2009; this study was performed from September 2009 to December 2009. Table 1 shows the number and mean age of the children in each class in the intervention and control groups. There was no significant difference in children' and mothers' ages between the intervention group and the control group (*p* = 0.28 and 0.86, respectively). None of the test scores showed a statistically significant difference between the two groups at Assessment 1 (Table 2, 3 and 4), indicating that they could be regarded as equivalent.

**Table 1:** Class profiles of number and mean age.

Class	C1	C2	C3	C4	I1	I2	I3	I4	I5
N	28	26	21	30	23	21	23	20	27
Mean age	4.38	4.17	5.31	5.30	4.33	4.27	4.19	5.29	5.34
(SD)	(0.47)	(0.33)	(0.44)	(0.43)	(0.44)	(0.41)	(0.35)	(0.43)	(0.45)

Class: C means control group, and I means intervention group.

N means the number of the children in each class. SD means the standard deviation of the children's mean age in each class.

**Table 2:** Results of the Parenting Stress Index: the child domain.

		Assessment 1 Mean (SD)			Assessment 2 Mean (SD)		pre-post change Mean (SD)			Effect size (95% CI)	within -class variation	between -class variation	Diagonal CSR	ICC	*p* value
	Subscale	IG	CG	P	IG	CG	IG	CG	Total						
Child domain	Reinforces parent	11.70	11.22	0.21	11.02	12.21	−0.68	0.99	0.12	0.53	3.18	6.81	9.99	0.32	0.39
		(2.89)	(2.76)		(2.73)	(3.65)	(3.06)	(3.24)	(3.15)	(0.40–0.67)					
	Mood	16.56	16.62	0.92	15.48	16.50	−1.08	−0.12	−0.62	0.27	0.10	12.83	12.93	0.01	0.04*
		(4.95)	(4.35)		(4.75)	(4.63)	(3.37)	(3.82)	(3.58)	(0.13–0.40)					
	Demandingness	9.80	9.37	0.35	8.77	9.72	−1.03	0.35	−0.37	0.54	1.27	5.15	6.42	0.20	0.12
		(3.55)	(3.24)		(3.11)	(3.41)	(2.46)	(2.60)	(2.52)	(0.41–0.68)					
	Distractibility/Hyperactivity	13.05	13.32	0.58	12.69	12.58	−0.36	−0.74	−0.54	0.14	−0.03	7.35	7.33	0.00	0.29
		(3.53)	(3.59)		(3.65)	(3.48)	(2.46)	(2.96)	(2.70)	(0.01–0.27)					
	Adaptability to people	11.02	11.54	0.26	10.56	10.79	−0.46	−0.75	−0.60	0.12	0.10	7.49	7.59	0.01	0.31
		(3.19)	(3.54)		(3.85)	(3.45)	(2.58)	(2.93)	(2.75)	(0.03–0.24)					
	Acceptability to parent	7.99	8.04	0.90	7.41	7.95	−0.58	−0.09	−0.35	0.20	−0.02	6.15	6.13	0.00	0.28
		(2.64)	(2.72)		(2.76)	(2.89)	(2.40)	(2.56)	(2.48)	(0.06–0.33)					
	Adaptability to things	7.69	7.88	0.54	7.26	7.74	−0.43	−0.14	−0.29	0.14	−0.02	4.49	4.47	0.00	0.02*
		(2.34)	(2.23)		(2.42)	(2.49)	(2.07)	(2.16)	(2.11)	(0.00–0.27)					
	Total score of child domain	78.11	77.91	0.92	73.20	77.50	−4.91	−0.41	−2.75	0.36	2.28	149.45	151.73	0.02	0.04*
		(16.79)	(14.03)		(16.25)	(17.93)	(10.73)	(13.82)	(12.21)	(0.23–0.50)					

* indicates statistically significant differences in Assessment 2 relative to Assessment 1 (p < 0.05; linear mixed model analysis). P means the value of the two sample t-tests between the subscale means of the intervention and the control group at Assessment 1. *p* value means the value of the linear mixed model analysis between the two groups in Assessment 1-Assessment 2 changes. Effect size means the value of Cohen's d statistic, which is defined as the the difference between two means divided by a standard deviation for the data. IG = the intervention group, CG = the control group, SD = standard deviation, CI = confidence interval, CSR = compound symmetry correlation, ICC = intraclass correlation.

**Table 3:** Results of the Parenting Stress Index: the parent domain.

		Assessment 1 Mean (SD)			Assessment 2 Mean (SD)		pre-post change Mean (SD)			Effect size (95% CI)	within -class variation	between -class variation	Diagonal CSR	ICC	*p* value
	Subscale	IG	CG	P	IG	CG	IG	CG	Total						
Parent domain	Role restriction	18.46	18.76	0.67	18.84	18.70	0.38	−0.06	0.17	0.11	0.03	15.56	15.59	0.00	0.49
		(5.73)	(4.84)		(5.19)	(5.29)	(3.50)	(4.39)	(3.93)	(0.02–0.25)					
	Isolation	14.55	14.72	0.79	14.46	15.14	−0.09	0.42	0.15	0.15	0.04	10.99	11.02	0.00	0.08
		(4.60)	(4.59)		(4.61)	(4.66)	(3.00)	(3.63)	(3.30)	(0.02–0.29)					
	Spouse	11.10	11.57	0.41	11.46	11.30	0.36	−0.27	0.06	0.20	−0.04	10.27	10.23	0.00	0.13
		(4.60)	(3.82)		(4.07)	(4.13)	(3.18)	(3.22)	(3.20)	(0.06–0.33)					
	Competence	21.13	21.05	0.88	20.29	20.99	−0.84	−0.06	−0.47	0.30	0.00	6.64	6.64	0.00	0.05*
		(3.63)	(3.55)		(3.63)	(3.89)	(2.51)	(2.65)	(2.58)	(0.17–0.44)					
	Depression	9.89	9.71	0.68	9.13	9.63	−0.76	−0.08	−0.43	0.26	0.00	7.01	7.01	0.00	0.22
		(3.21)	(3.01)		(3.40)	(3.40)	(2.45)	(2.85)	(2.65)	(0.12–0.39)					
	Attachment	6.48	6.04	0.14	5.77	6.26	−0.71	0.22	−0.26	0.46	0.06	4.10	4.17	0.02	0.49
		(2.28)	(2.07)		(2.06)	(2.08)	(1.94)	(2.14)	(2.04)	(0.32–0.59)					
	Health	7.22	7.12	0.77	6.82	7.35	−0.40	0.23	−0.10	0.28	−0.02	5.09	5.07	0.00	0.07
		(2.44)	(2.50)		(2.56)	(2.54)	(2.19)	(2.31)	(2.25)	(0.14–0.41)					
	Total score of parent domain	96.86	96.54	0.90	94.01	97.35	−2.85	0.81	−1.10	0.30	−0.68	152.79	152.12	0.00	0.07
		(20.23)	(18.47)		(20.68)	(20.91)	(11.31)	(13.37)	(12.30)	(0.16–0.43)					

See Table 2 legend.

**Table 4:** Results of the Goodenough Draw-a-man Intelligence Test and the New S-S Intelligence Test.

		Assessment 1 Mean (SD)			Assessment 2 Mean (SD)		pre-post change (SD)			Effect size (95% CI)	within -class variation	between -class variation	Diagonal CSR	ICC	*p* value
	Subscale	IG	CG	P	IG	CG	IG	CG	Total						
The DAM	Intelligence Quotient	89.56	87.25	0.34	94.61	87.96	5.05	0.71	2.96	0.26	−0.85	218.21	217.36	0.00	0.03*
		(17.60)	(18.09)		(20.95)	(19.78)	(14.83)	(13.16)	(14.03)	(0.12–0.39)					
The NS-SIT	Understanding relationships between things	4.46	4.08	0.39	7.41	6.46	2.95	2.33	2.65	0.21	0.07	7.64	7.71	0.01	0.99
		(3.30)	(3.31)		(2.50)	(3.27)	(2.84)	(2.71)	(2.78)	(0.08–0.35)					
	Counting and comparing the numbers	1.87	1.74	0.56	2.63	2.57	0.76	0.82	0.79	0.02	−0.12	3.05	2.93	−0.04	0.87
		(1.66)	(1.54)		(1.90)	(1.96)	(1.62)	(1.80)	(1.71)	(−0.11–0.16)					
	Calculation	3.61	3.28	0.19	4.42	4.07	0.81	0.79	0.80	0.01	0.07	1.93	1.99	0.03	0.93
		(1.79)	(1.97)		(1.38)	(1.78)	(1.36)	(1.46)	(1.41)	(−0.12–0.15)					
	Completion of the pictures	2.65	2.58	0.85	5.75	4.95	3.10	2.02	2.58	0.15	1.06	3.36	4.41	0.24	0.32
		(2.75)	(2.59)		(1.99)	(2.17)	(2.11)	(1.97)	(2.04)	(0.02–0.29)					
	Working memory	3.64	3.77	0.52	4.35	4.03	0.71	0.23	0.48	0.32	0.33	2.28	2.61	0.13	0.02*
		(1.57)	(1.46)		(1.31)	(1.57)	(1.52)	(1.71)	(1.61)	(0.19–0.45)					
	Processing speed	3.4	3.57	0.72	7.26	6.14	3.86	2.20	3.06	0.29	3.63	5.55	9.17	0.40	0.03*
		(3.48)	(3.46)		(1.56)	(2.53)	(2.91)	(2.89)	(2.90)	(0.16–0.43)					
	Total score	21.45	20.34	0.42	29.45	26.92	8.00	6.58	7.32	0.24	0.15	36.00	36.14	0.00	0.08
		(9.69)	(10.52)		(9.09)	(11.08)	(5.69)	(6.34)	(6.00)	(0.10–0.37)					

See Table 2 legend.

### *1.* Child and mother psychosocial problems related to parenting stress (Table 2, 3 and Appendix S2)

“Total score of the child domain” which was one of the primary outcomes of child and mother psychosocial problems related to parenting stress showed significant improvement in the intervention group compared to the control group (*p* = 0.03, effect size (ES) = 0.31 [95% confidence interval (CI): 0.18–0.45]). Another primary outcome, “Total score of the parent domain” showed no significant improvement in this program (*p* = 0.07, ES = 0.30 [95% CI: 0.16–0.43]). The analyses of the secondary outcomes revealed significant improvement in the intervention group compared with the control group in the child domain subscales of “Mood” (*p* = 0.04, ES = 0.25 [95% CI: 0.11–0.38]), “Adaptability to things” (*p = *0.02, ES = 0.29 [95% CI: 0.15–0.42]). In the parent domain, “Competence” showed significant improvement in the intervention group compared to the control group (*p* = 0.05, ES = 0.22 [95% CI: 0.09–0.36]). The results of all subscales showed larger improvements in the intervention group than in the control group, with the exception of two subscales in the child domain, “distractibility/hyperactivity” (*p* = 0.29, ES = 0.14 [95% CI: 0.01–0.27]) and “adaptability to people” (*p* = 0.31, ES = 0.12 [95% CI: 0.03–0.24]); and two subscales in the parent domain, “role restriction” (*p* = 0.49, ES = 0.11 [95% CI: 0.02–0.25]) and “spouse” (*p* = 0.13, ES = 0.20 [95% CI: 0.06–0.33]). According to these two subscales, the scores of the intervention group increased, and those of the control group decreased.

### 2. Child cognitive abilities (Table 4 and Appendix S3)

Children's IQ in the DAM, the primary assessment of the children's cognitive abilities, was significantly enhanced compared with the control group (*p* = 0.03, ES = 0.26 [95% CI: 0.12–0.39]). In the NS-SSIT, the scores of the intervention group significantly improved in “Working memory” (*p* = 0.02, ES = 0.32 [95% CI: 0.19–0.45]) and “Processing speed” (*p* = 0.03, ES = 0.29 [95% CI: 0.16–0.43]) compared with those of the control group.

### 3. Fidelity assessment (Figure 2)

**Figure 2:**
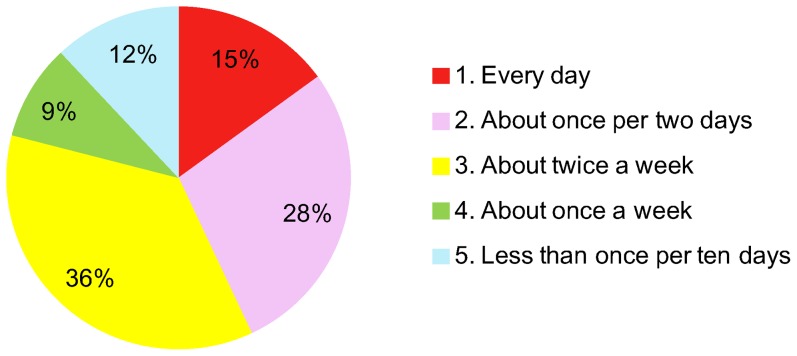
**Results** **of the fidelity assessment of the program in the intervention group.**

The results of the fidelity assessment in the intervention group are listed below.

1. Every day: 15%, 2. About once every two days: 29%, 3. About twice a week: 37%, 4. About once a week: 9%, 5. Less than once every ten days: 14%

## Discussion

The present study provides evidence that our intervention program may ameliorate psychosocial problems in children related to parenting stress and increase the children's cognitive abilities. These findings suggest that this program may enhance psychosocial development in children. The program did not significantly affect psychosocial problems of the mothers related to parenting stress; however, the results suggest that the program may improve maternal confidence in parenting.

### Child and mother psychosocial problems related to parenting stress

The improvement in “Total score of the child domain” led us to propose that this program may ameliorate child psychosocial problems related to parenting stress, since high scores in this subscale are associated with children who display some psychosocial problems that make it difficult for parents to fulfill their parenting roles [12]. We believe that mother–child play activities focusing on maternal emotional sensitivity enhanced the parent-child interactions and their relationship, and thus improved the child's dysfunctional behavior. This improvement might be derived from the mothers' changes, i.e. they would have become able to accept their children's psychological and behavioral problems and better able affirm their children in this program. Parenting stress is significantly related to child psychosocial development and problems, including social competence, internalizing behaviors (i.e. depressive, anxious, isolative, and dependent behaviors) and externalizing behaviors (i.e. angry, aggressive, egotistical, and oppositional behaviors) [38]. We also suggest that this program may improve child psychosocial development and its related problems.

This program did not show significant improvement in the primary outcome of mother psychosocial problems, “Total score of the parent domain”. The results suggest that this program seems not to be effective for improving mother psychosocial problem related to parenting stress. This suggests that our program does not improve the mother's psychological status and parenting stress, perhaps because the positive impact of the intervention was primarily on the mother–child interaction and had less influence on the mother's psychosocial problems. During the early years, parents are the primary source of a child's sense of self-worth and the principle teachers of effective self-control of psychosocial behaviors [13]. Mothers have relationships other than that with their child, and these relationships can be a source of psychosocial problems. This may explain why our program had a significant effect on the total score of the child domain, but not the parent domain. This suggests that approaches that are more direct than encouraging mother–child interaction are needed to reduce the mothers' psychosocial problems related to parenting stress.

The children's improvement in “Adaptability to things” suggests that the intervention encouraged children to develop an enterprising and open attitude to the outside world. These changes were most likely the result of increased attachment in the mother–child relationship. Children who have a positive attachment to their mothers internalize representations of the attachment and apply them to other relationships. In attachment theory, this is called the internal working model [39]. Children come to believe that the world is safe and that their mothers will always protect them. Attachment theory refers to this as a “secure base” [40]. According to Bowlby [41], children are likely to explore their environments when they feel sufficiently protected and comforted by their mother's presence [40]. Children's confidence in this secure base determines the extent to which they feel free to explore, play, and learn. The sense of a secure caregiver allows exploration and enables the child to regulate affect, manage behavior, achieve autonomy, and develop a sense of self [42]. Improvement in “Adaptability to things” subscale indicated that our program helped the children develop a more secure base and an open, accepting attitude toward the world. Bowlby [43] believed that the internal working model gradually developed during childhood and became stable and rigid as children grew up, suggesting plasticity in the internal working model during childhood [44]. We suggest that an attachment-based intervention in childhood, such as that offered in this program, has a positive impact on the development of the internal working model. The internal working model for attachment formed in early childhood has been reported to be stable over time and predictive of several important outcomes during childhood and adolescence, such as social competence among peers, the ability to cope with stress, psychological health, and school adjustment [45], [46]. The Minnesota Parent–Child Project found that attachment status in infancy predicted significant features of personality several years later, including risk for anxiety disorders in adolescence and other variables predictive of difficulty later in life. [47]. Based on the finding that the intervention in the present study strengthened the mother–child attachment, we surmise that our program can prevent psychological and psychiatric problems later in the child's lives.

In contrast, our program did not significantly improve “Adaptability to people” score. This may be because the children played with their mothers, classmates, and teachers, but not with people they did not know. However, in the long term, our program may have a positive effect on children's relationships with people they do not know. Improvement in the Adaptability to things subscale may indicate that the children have developed a positive internal working model that has a positive effect on relationships with others. This hypothesis will be investigated in a follow-up study.

The significant improvement in the child domain “Mood” score led us to infer that the program may improve the children's mental health, as high scores on this subscale are associated with affective dysfunction [25]. This may be the effect of a positive mother–child interaction; the improvement in the mother's attitude toward her child makes it easier for her to accept the child's behavioral problems. Alternatively, the improvement in mood may have been the result of changes in the prefrontal cortex of the brain. Brain imaging studies have shown that mood disorders are related to prefrontal cortex function [48]. Takeuchi and colleagues [49] reported that cognitive training altered structural connectivity in the brain by increasing pruning and myelination, thus, improving cognitive ability. Because the play activities in our program were based on standard cognitive tasks associated with prefrontal cortex activation, the significant improvement in “Mood” may have been related to effects on the children's prefrontal function.

The subscale, “Competence” in the parent domain was significantly enhanced in the intervention group compared with the control group. Parents who have a limited range of child management skills show high scores on this subscale [50]. Furthermore, parents who do not find parenting to be as reinforcing as they had expected tend to have high scores on this subscale [51]. Improvement on this subscale may have been associated with our request that mothers focus on maternal sensitivity toward their children. Increased maternal sensitivity may have affected the children's responsiveness to their mothers, thus improving the mother–child relationship. In turn, the mothers, feeling the effect of their emotional sensitivity may have become more self-assured as parents. Our results suggest that this program can instill parenting confidence in the mother.

In the child domain, the score changes in “Distractibility/Hyperactivity” and “Adaptability to people” were larger in the control than in the intervention group. However, both groups showed improvement in these subscales, and the statistical analyses revealed no significant difference between the changes in these subscales demonstrated by the two groups.

Two parental domain subscales, “Role restriction” and “Spouse” changed in a different direction from the other subscales of the PSI. In these subscales, the control group score improved slightly, and the intervention group was somewhat higher. However, these changes were not statistically significant. “Role restriction” reflects the mother's sense of being controlled and dominated by her child's demands and needs [14]. The increased score in the intervention group may have indicated that the mothers felt restricted by the demands of our program; however, the intervention gave them the opportunity to play with their children in a way that enhanced the mother–child relationship. Parents who have a high score on “Spouse” lack the emotional and active support of the other parent in childcare [52]. The increase in the intervention group's score on this subscale may have been the result of not involving the fathers in the program. The mothers of the intervention group might have felt not being supported by their partners in the program, since the fathers were not involved. This finding suggests that family members in addition to the mother should be involved in the program.

### Child cognitive abilities

The results support the view that this program may enhance children's intelligence quotients (IQ) of the DAM. The IQ of the DAM is known to correlate highly with fluid intelligence [53]. Fluid intelligence is the capacity to think logically and solve problems in novel situations, independent of acquired knowledge. It is also the ability to analyze novel problems, the ability to identify the patterns and relationships that underpin these problems, and the extrapolation of these using logic [54]. It is a pivotal to our ability to adapt our thinking to new cognitive problems or situations [55]. It is also closely related to professional and educational success [56], [57], [58], especially in a complex and demanding environment [59]. Jaeggi and his colleagues revealed that adults' fluid intelligence can be improved by training [60]. Our results indicate that this mother and child play activity program can enhance children's fluid intelligence. Gray and his colleagues revealed that lateral prefrontal and parietal regions are related to abilities and performances of general fluid intelligence [61]. The play activities in this program were designed to associate with PFC activities based on previous neuroimaging studies. We believe that the program enhanced the children's general fluid intelligence partly because it was designed to be associated with PFC activities.

The results of the NS-SIT showed that the scores of the intervention group significantly improved in “Working memory” and “Processing speed” compared with the control group.

The improvement of WM in our program may have led to improvement of EF. This is because WM is one of the principal factors of EF. According to Miyake and colleagues, EF has three factors which correlate with one another: shifting of mental sets, monitoring and updating of WM, and inhibition of proponent responses [62]. Therefore, in our program, the enhancement of WM may have also improved other domains of EF, and thus EF as a whole. Much research has revealed that EF is deeply related to inhibitory control [6]. It has been proposed that inhibitory control plays a central role in fostering self-regulation [62], [63]. Inhibitory control is also proposed to play a critical role in academic learning, by promoting children's capacity to think about multiple dimensions of perspectives on a problem [64], [65]. Thus, EF capacities appear to play a central role in fostering the focused and rule-governed behavior that supports both cognitive and social-emotional adjustments [66]. These findings are consistent with the fact that dysfunction of EF causes emotional regulation and behavioral problems, including aggression, depression, and attention disorders [63]. On the basis of these findings, many preventive interventions were developed focused on supporting improved emotional self-regulation and problem-solving skills in which EF and the PFC functions play central roles [67]. We think that our program may promote children's emotional self-regulation and problem-solving abilities through enhancement of EF.

The resulting improvement in “Processing speed” suggests that the program may enhance the children's processing speed (PS). PS heavily influences WM because memory processing and storage is time related [68]. Faster PS allows more information to be processed in less time, thereby increasing the functional capacity of WM [69]. Since WM is a major factor of EF, the results lead us to conjecture that our program enhances EF by powering PS.

The results of the enhancement of children's WM and PS in our program also lead us to surmise that the program may lead to enhance children's fluid intelligence. In Fry and Hale's model, ability change in PS increases the functional capacity of WM, which in turn facilitates fluid intelligence [70]. The program enhanced WM and PS in the NS-SIT, as well as the intelligence quotient in the DAM, which is deeply related to fluid intelligence. We consider that our program may enhance children's fluid intelligence, increasing WM powered by PS.

Children's play activities are greatly affected by their social and cultural background [71]. We used the NS-SIT for the assessments of children's abilities in this study because the test was developed for people with a Japanese social and cultural background. The results lead us to believe that this program socially and culturally enhances child cognitive abilities.

Van IJzendoorn revealed that child cognitive abilities are correlated with both attachment and communication [72]. We suggest that enhancement of cognitive abilities through our program improves communication and may improve feelings of attachment in mother and child. Cognitive and social abilities are essential to one another [73]. Therefore, the results lead us to surmise that enhancement of children's mental health and their cognitive abilities may have had mutual enhancement effects on each other. Berk and Winsler proposed that good scaffolding enhances child cognitive and social abilities [21]. We give rise to the view that, by strengthening scaffolding, our program has enhanced improved mental health and child cognitive abilities.

The scores of the control group improved more than did those of the intervention group in “Calculation” of the NS-SIT. However, the *p* value of the statistical analysis revealed that the difference between the two groups was not significant.

### Generalizability

This program only takes about ten minutes per day. Without special training or education, mothers and children can enjoy the program. The load of performing this program is reasonably light. We believe that our program has the characteristics of a good public health program for improving child psychosocial problems related to parenting stress and thus preventing psychological and psychiatric problems and enhancing child cognitive abilities.

There are many academic areas relating to children: brain science, preschool pedagogy, developmental psychology, child and maternal psychiatry, and so on. Each area has made remarkable advances. We think the integration of many academic areas is necessary for developing new viewpoints and stimulating progress in the various research fields relating to children. Such advancements would greatly benefit children and their families. As far as we know, this is the first investigation which examined a program for improving child psychosocial problems related to parenting stress and for enhancing child cognitive abilities using play activities by integrating brain science, preschool pedagogy, developmental psychology and child and maternal psychiatry.

### Limitations and future directions of the current study

Although the play activities in our program were based on the standard cognitive tasks used in previous neuroimaging studies which are associated with PFC functions, we did not examine, using neuroimaging techniques, whether or not our program is really associated with PFC functions. Further investigations will be needed to demonstrate children's brain activities during participation in our program. It is also still unclear which factor contributed more to the effects of our program: the cognitive training or the positive parenting through the play activities. Our play activity program, which includes the characteristics of parents' sensitive emotional responses and cognitive training for children, can be regarded as an educational program package. Sensitive emotional response couldn't be excluded from our play activity program because it must exist in parent and child play activity programs from the viewpoint of preschool education. Therefore, sole examination of either the effects of cognitive training or parents' sensitive emotional responses could not be included in our present study.

The participants in this study were recruited from the middle and the upper levels of a kindergarten, which determined the sample size. To arrive at a realistic estimate of the power of the study, we calculated the effect size to be 0.45 based on a previous meta-analysis [16]. Using *t*-tests with 80% power at the two-sided significance level of 0.05, we determined that 158 pairs would be needed to detect a 0.45 standardized mean difference between the two groups in terms of the primary outcomes of Assessments 1 and 2. Because the mean number of children in this kindergarten class was 24.3, we used 24 as the mean class size for this estimate. Intraclass correlation (ICC) values are generally between 0.01 and 0.02 for human studies [74]. If we employed 0.01 as the ICC, the design effect [75] would be 1.23. When the sample size was adjusted according to the design effect, 195 child–mother pairs were needed. Assuming a dropout rate of 20 pairs (about 10%), the study would have needed a final sample size of 215 pairs. Thus, recruitment of the entire middle and the upper levels of the Wakakusa kindergarten class was appropriate. If we had employed 0.02 for the ICC, the design effect would have been 1.46. When the sample size was adjusted according to the design effect, 231 child–mother pairs would have been needed, yielding a sample a little larger than that of this study (219). With respect to the primary outcome, the ICCs for the total scores of the child and parent domains of the PSI and of the IQ of the DAM were 0.02, 0.01, and 0.00, respectively. The ICC of the PSI child domain was 0.02, which could have rendered the power of this study insufficient. However, we observed a significant difference between the intervention and control groups in the outcome measures. Thus, this study can be regarded as having sufficient power for the primary outcomes. As shown in Tables 2, 3 and 4, most of the secondary-outcome ICCs were less than or approximately equal to 0.01. However, several secondary outcomes had large ICCs (i.e., “Reinforces parent” and “Demandingness” in the PSI and “Counting and comparing the numbers,” “Calculation,” “Completion of the pictures,” “Working memory,” and “Processing speed” in the NS-SIT). It is not clear which factors contributed to these high ICCs and how they did so. In terms of the subscales of the PSI, the high ICCs for reinforces parent and demandingness may be attributable to the environment of the kindergarten class, including the interactions among children in the class, the effects of the community involving the class mother, and the teachers' educational efforts. Regarding the subscales or the NS-SIT, the high ICCs for counting and comparing the numbers, completion of the pictures, working memory, and processing speed may be attributable to the effect of performing these tests in the classroom (i.e., the examiner's orientation and the class environment); these factors may also be relevant to the results of the PSI subscales. We considered the notion that the sample be large enough to have power for all the secondary outcomes to be unrealistic and unnecessary because this study was an exploratory examination. However, further research will be needed to examine how these external factors affect ICCs and program effectiveness.

The results of the assessment of participants' fidelity to this program revealed that about 20% of the participants performed the program at the frequency of once a week or less than once every ten days. Those participants can be regarded as having not performed the program. Since we did not ask the participants about their living conditions in the questionnaire, we cannot correctly discuss the reason why these participants could not perform the program. However, we guess that it is partly because some mothers work and could not set aside enough time for the program. How we can reach out to such people also should be investigated in the future.

The present study involved multiple testing because the assessment tests used, the PSI and the NS-SIT, have several subscales. Multiple testing increases the risk of a type 1 error [76]. We did not correct for multiple comparisons in the analyses because it was our intention to conduct an exploratory examination of which stress factors measured by the PSI and which cognitive abilities measured by the NS-SIT were improved by our program. Based on the results of the present study, further research should be conducted to assess specific subscales using multiple comparisons [77].

The measures of child and mother psychosocial problems related to parenting stress used in our study were based solely on the mothers' reports, and no independent observations were performed. It is not clear what aspect of the intervention was responsible for this program's effectiveness. We suggest, as mentioned above, that enhanced mother-child attachment and the cognitive abilities of children which were related to prefrontal functions played important roles in our results because those were the focuses of this program. Further investigations will be needed using measures of maternal sensitivity and behavioral changes, as well as observation measures.

This emotional sensitivity focused program targeted solely on the mother–child relationship, and revealed the program effectiveness for the improvements of the child psychosocial problems related to parenting stress and parenting confidence. It can be regarded as a parenting support program. Previous studies indicated that parenting support programs involving fathers can impact on their parenting behaviors [78], child development [78], [79] and also family system [80]. Future studies involving other family members and investigating changes in family system with this program are needed.

We developed this program using various types of play activities designed to enhance a variety of cognitive abilities rather than specific abilities. It is unclear to what extent this specific play activity intervention was responsible for the observed changes, or whether any daily 10-min play activity session would have been effective. Moreover, the preparatory run-through with teachers in the kindergarten may have been responsible for the changes. We proposed this program as an educational package, collaboration between the kindergartens and the families. Thus, we cannot distinguish between the contribution of the family and that of the kindergarten to our results. Further research is needed to identify which play activities were responsible for improving which cognitive abilities.

The fidelity assessment suggested that only a minority of participants performed the intervention as planned (44% at least once every 2 days). However, the effect in terms of reducing parenting stress and enhancing the children's cognitive abilities was large. It may be that an improvement in emotionally sensitive parenting, rather than the daily play activities, was responsible for the reduction in parenting stress. Furthermore, performing the play activities daily in the kindergarten may be as important in increasing the child's cognitive ability as practicing them at home. The next step in our investigation is to identify the factors underlying the effectiveness of our program.

The long term effects of the program are still under investigation. We intend to perform a two-year follow-up study. Our program was effective for typically developed children and their mothers. On the other hand, the effect of the program on mothers and children who have risk factors in their attachment to one another and in their mental health is still unknown. Further investigation should be conducted in the future.

## Conclusion

We propose a new play activity intervention program for mother and child integrating interdisciplinary areas for children, namely brain science, developmental psychology, preschool pedagogy and child and maternal psychiatry. To identify the effects of the program, we conducted a cluster randomized controlled trial. The results suggested that our intervention program may ameliorate children's psychosocial problems related to parenting stress as well as their cognitive abilities. It was also suggested that the program may help mother and child to develop a more positive attachment to each other and improve children's mood. In addition, the program may increase the mothers' self-assurance as parents. The present study reveals a new potential early childhood intervention using interdisciplinary knowledge to enhance child psychosocial development and to decrease parenting stress.

## Supporting Information

Checklist S1 **CONSORT Checklist.**
(DOC)

Protocol S1 **Trial Protocol.**
(DOC)

Appendix S1 **The details of the program.**
(PDF)

Appendix S2 **The Parenting Stress Index mean score change in each class in the intervention and control groups.**
(PDF)

Appendix S3 **The Goodenough Draw-a-man Intelligence Test and the New S-S Intelligence Test mean score change in each class in the intervention and control groups.**
(PDF)
