# Supplementary material for: A New Mother-Child Play Activity Program to Decrease Parenting Stress and Improve Child Cognitive Abilities: A Cluster Randomized Controlled Trial
Source: PLoS One. 2012 Jul 27;7(7):e38238. doi: 10.1371/journal.pone.0038238 (PMC3407189; doi:10.1371/journal.pone.0038238)
Supplement: Protocol S1 — Trial Protocol. (DOC) [file pone.0038238.s002.doc]

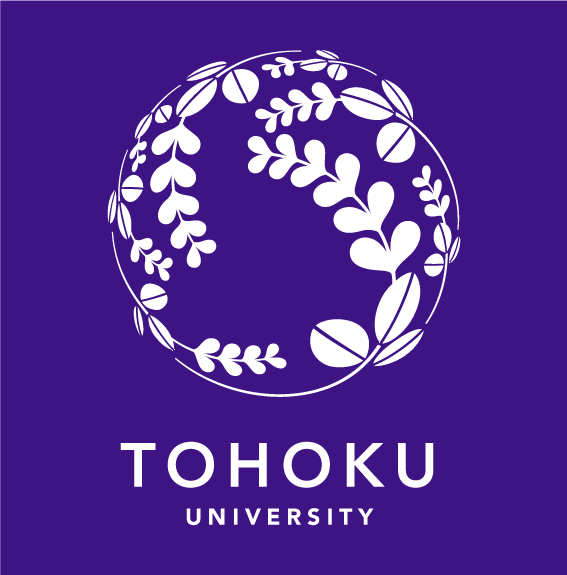


**脳を育む遊びによる親子の愛着形成及び認知力向上の検証**

MAN004　Ver.1.2

作成者

立花良之　　　　　　　　　　　　　　　　　　　日付：2009年7月1日

川島隆太　　　　　　　　　　　　　　　　　　　日付：2009年7月1日

研究代表者　　　　川島隆太

東北大学加齢医学研究所スマート・エイジング国際共同研究センター

〒980-8575　宮城県仙台市青葉区星陵町4-1

TEL　022‐717-7988 FAX 022-717-7988

E-mail : ryuta@idac.tohoku.ac.jp

研究事務　　　　　　東北大学加齢医学研究所スマート・エイジング国際共同研究センター

〒980-8575　宮城県仙台市青葉区星陵町4-1

TEL　022‐717-7988 FAX 022-717-7988

E-mail : tatibana@idac.tohoku.ac.jp

受付番号：　2009-409

プロトコル確定日：2009年7月1日

**0　概要**

- 研究目的：親子の遊びのプログラムが子どもの認知発達やメンタルヘルスへの効果を検証する
- 対象者適格規準：本研究の趣旨を理解し参加に同意したわかくさ幼稚園在籍の児童及びその母親
- 対象者除外基準：本プログラムを行えないような臨床的に重篤な病気や障害を子どもか母親が持っていること。
- 生活介入計画：親子で行う遊びを月に4種類用意し、親子で1日1種類の遊びを10分程度楽しむことを1週間に5回、3か月間行うものとした。この際親に対しては、遊びを通し子どものありのままの存在を受けとめ積極的に子どもをほめたり認めたりしてもらう。
- エンドポイント：

こどもの認知発達

主要エンドポイント: グッドイナフ人物画知能検査　知能指数

副次エンドポイント：新S-S知能検査の下位項目

子どものメンタルヘルス

主要エンドポイント：育児ストレスインデックス　「こどもの側面」

副次エンドポイント：育児ストレスインデックス　子どもの側面の下位項目

親のメンタルヘルス

主要エンドポイント：育児ストレスインデックス「親の側面」

副次エンドポイント：育児ストレスインデックス　親の側面の下位項目

- 目標対象者数（サンプルサイズ）：220名
- 実施予定期間：2009年9月～2009年12月
- 問い合わせ先：東北大学加齢医学研究所スマートエイジング国際共同研究センター

〒980-8575 宮城県仙台市青葉区星陵町4－1

TEL 022-717-7988

E-mail: tatibana@idac.tohoku.ac.jp

**1. 目的**

本研究では、脳科学、発達心理学、幼児教育学、乳幼児精神医学の知見を統合し、それらの理論を背景とし、また、前頭前野の活動と関連する認知機能課題から考案した遊びのプログラムを作成する。この遊びのプログラムは、子どもの発達の足場作りに役立つように配慮され、また、親が子どもの情緒に敏感に反応することを重要視する。よって、このプログラムに親子が参加することで、参加していない場合に比べて、子どもの認知発達を促進し、また子どものメンタルヘルスも向上させると予想する。本研究の目的はこの遊びのプログラムが、子どもの認知発達を促進させ、メンタルヘルスを向上させる効果を持つとの仮説を検証することである。

エンドポイント：

こどもの認知発達

主要エンドポイント: グッドイナフ人物画知能検査　知能指数

副次エンドポイント：新S-S知能検査の下位項目

子どものメンタルヘルス

主要エンドポイント：育児ストレスインデックス　「こどもの側面」

副次エンドポイント：育児ストレスインデックス　子どもの側面の下位項目

親のメンタルヘルス

主要エンドポイント：育児ストレスインデックス「親の側面」

副次エンドポイント：育児ストレスインデックス　親の側面の下位項目

**2. 背景**

2.1 脳科学の観点から

　前頭前野は、他者とのコミュニケーションや、物事の計画を立てること、推論すること、言葉を理解することなど、人が日常生活を営む上で重要な様々な機能を担っている[1]。この前頭前野の機能は、幼少期から思春期にかけて著しく発達する [1]。近年、脳科学研究で「脳を育む」という領域に関心が高まり、その中でも前頭前野を健全に育む重要性が注目されている[2]。脳画像研究により、ある認知課題を行うとそれに関連する脳領域の脳血流や脳代謝が亢進することが分かっている[3]。Kawashimaらは、Alzheimer型老年認知症の患者に読み書き計算の生活介入を行うことで認知症の症状の改善の効果がある[4]ことや、健常高齢者に同様の生活介入を行うことで認知力を向上させる効果がある[5]ことを示した。我々は、前頭前野の活動に関連するような生活介入が、子どもにも良い影響を及ぼすのではないかと考えた。

　本研究において、我々は、幼稚園の児童を生活介入プログラムのターゲットとする。前頭前野の機能の中でとりわけ重要な働きをもつ実行機能[6]は、3歳から5歳に急激に発達する[7]ため、幼少期の多くの生活介入研究が、実行機能をターゲットとしている[8]。3歳から5歳は幼稚園の時期に当たるが、この時期に実行機能を育むことは、子どもの健全な発達の促進を考える上でとりわけ重要である[9]。前頭前野の機能に関連した認知課題についての脳画像研究は数多く行われている。脳画像研究で用いられている認知課題には、たとえば空間ワーキングメモリ課題[10]や語流暢性課題[11]など、形式、難易度を調整すれば幼稚園生でも実施可能なものが多い。そこで我々は先行する脳画像研究に基づき、生活介入プログラムを作成する。

2.2 発達心理学、幼児教育学の観点から

本研究では、子どもの発達を促すものとして、親子の遊びをプログラムに取りいれる。さらに、親子の遊びのプログラムを考える上で、最近接発達領域と「足場作り」に着目する。

最近接発達領域とは、「子どもが自力で達成可能な水準と大人の援助を受けて達成可能になる水準の間のこと」であり[12]、Vygotskyが提唱した概念である。Vygotskyによれば、教育の役割は、最近接発達領域に働きかけ、大人の適切な支援によって子どもたちが達成することができる活動の経験を子どもたちにもたらすことである[12]。よって、我々はこの親子の遊びのプログラムを通して、子どもの最近接発達領域に働きかけ、それにより認知発達を促進することを目指す。

我々は、また、教育学でいわれている発達の足場作りの概念にも着目する。「足場作り」とは最近接発達領域において効果的な教授と学習の相互作用を土台として子どものより良い発達へつなげることの隠喩である。最近接発達領域をとらえることで効果的な親子の相互作用により子どもの認知発達や社会性の発達を促進しうることがわかっている[13]。以上より、我々は子どもの最近接発達領域を捉えるような遊びのプログラムを考案する。

2.3 乳幼児精神医学の観点から

　遊びを通して良好な愛着を促進することにより子どものメンタルヘルスを向上することを、本プログラムの目的のひとつとする。なぜなら、親子が一緒に遊ぶように促すことが子どものメンタルヘルスを改善する手段として役立つからである[14,15]。また、プログラム作成にあたって、親子の愛着促進についても重視する。大人が子どもとの共同作業において、楽しく受容的であり、適切に子どもをほめたり努力や能力を認めてあげれば、子どもの物事に取り組む意欲や、物事をひとりで行おうとする積極的な姿勢を伸ばすことができる[16]が、これらの態度は、親子の良好な愛着と非常に深く関わる[17]からである。愛着とは、特定の他者との間に築く緊密な情緒的結びつきのことで、良好な愛着は親子のメンタルヘルスにきわめて重要である[18]。愛着についての体系的な理論である愛着理論[19]を提唱したBowlbyは、愛着に基づく関係性が子どもたちの探索行動や遊びの発達のための主要な基盤を形成するとした[20]。愛着理論において、乳幼児期の正常な愛着は、人が健康に発達する上で重要であるとされている。愛着を形成した子どもは、やがて常に親と一緒にいなくても、親との愛着表象が自分の中に取り込まれることにより、安心感を得るようになる。そして、親の居場所を中心として、探索活動に熱中するようになる。このとき、安全が保障されていると感じる心理的基盤を「心の安全基地」という[20]。Bowlbyは生態行動学の観点から、子どもには生得的に環境を探索しようとする行動傾向があり、親との間に安定的な愛着関係が成立しているときには、積極的な探索行動を行うことができるが、不安定な愛着関係にあるときには十分な探索行動を行えないことを指摘した。探索行動をしている自分をいつでも見守り、そして疲れ果てたり、失敗し傷ついてもどってきたりしても暖かく迎え癒してくれる心理的な安全基地があれば、子どもは社会に対して積極的な姿勢を持つことができるとした。また、Bowlbyは、幼少期の愛着表象が後の人生の対人関係パターンに大きな影響を及ぼすとした[21]。親子を子どもが幼少期から青年期になるまで追跡したMinnesota Parent-Child Projectによれば、幼少期の愛着は後の人生における性格の重要な予測因子となり、青年期の不安障害のリスクとも関係することがわかっている[22]。子どもの幼少期において、親は子どもの自尊心や対人関係を営む力の発達の主要な源である[23]。子どもの不安定な愛着を改善したり予防したりするために、母親の敏感性を高める支持的な介入や母親に働きかける洞察志向の心理療法など、親子の生活介入プログラムの研究が多く行われている[24,25]。それらのメタアナリシスの結果として、母親の子どもに対する敏感かつ肯定的な情緒応答性を高めること、セッションが多くないなど介入プログラムにおける親の負担が少ないことが、愛着を深めるのに効果ある生活介入研究の特徴であることがわかっている[26]。また、行動療法の観点から、育児の中で子どもがうまくできたことをその場でほめて認めてあげることが、子どもの問題行動の改善や、メンタルヘルスの向上に効果があることがわかっている[27]。「親の子どもに対する敏感かつ肯定的な情緒応答性」とは、「子どもができたことを敏感に受け止め、子どもをほめて認めてあげること」とも捉えることができる。本プログラムにおいて、親の子どもに対する情緒応答性を向上させるため、子どもをほめて認めてあげることを強調した。また、親に対する負担を少なくするため、プログラムは親子で楽しく行え、実施することが親子にとって負担に思わないような内容になるように配慮する。

**3. 薬剤や器具の情報**

本研究では特別の薬剤や器具を用いない。

**4. 本試験で用いる規準・定義**

本研究では健常児及びその親を対象とするため、特記事項なし。

**5. 参加者選択規準**

以下の適格規準を全て満たし、かつ以下の除外規準のいずれにも該当しない親子を、本研究の対象とする。

**5.1. 適格規準**

わかくさ幼稚園に在籍する児童及びその母親で、本研究参加への同意書に同意・署名した者

**5.2. 除外規準**

本プログラムを行えないような臨床的に重篤な病気や障害を子どもか母親が持っていること。

**6. 登録・割り付け**

**6.1. 登録手順**

東北大学医学部倫理審査委員会にて、プロトコル、参加者への説明文書・同意文書の審査後、UMIN臨床試験登録システムにおいて、臨床試験登録を行う。

**6.2. 割り付け方法**

参加者を知らない無作為割り付けマネージャーが、封筒法に基づき幼稚園のクラス単位で無作為割り付けを行う。各々のクラスに対して番号が付され、その番号は介入群か比較群のどちらかに割り振られる。番号の割り付け表を、幼稚園教諭が無作為割り付けマネージャーから渡され、それに基づき、参加者を介入群か比較群に分ける。無作為割り付けマネージャーが無作為割り付けを行う場所は、幼稚園教諭が参加者を実際に2群に分ける場所とは別である。

**7.生活介入実施計画**

**7.1. 生活介入プログラムの内容**

遊びのプログラムは、子どもの好む遊びや、できるレベルを熟知した幼稚園教諭と脳科学研究者・児童精神科医で作成する。遊びの内容は、先行する脳画像研究で明らかとなっている前頭前野の活動に関わるような標準的な認知課題を基にする。これらの遊びの中から、ターゲットとする認知機能が異なるような遊びを1か月に4種類選び、親子で遊んでもらうこととする。また、子どもが親に家で遊びを教える形をとるが、正確に遊びの内容が親に伝わるように、１か月に1枚、4種類の遊びについて記載したプリントを配布する。そのプリントには4種類の遊びに4色の色を対応させる。それぞれの色について5枚ずつのシールとその月のカレンダーを各家庭に配布し、親子で遊んだ後に子どもがその遊びに対応する色のシールを、カレンダーのシートに張ってもらうこととして、取り組む遊びの種類が偏らず、均等に遊べるようにする。尚、何の遊びをどの日に行うかについては、各家庭で子どもに任せる。BerkとWinslerは、子どもの発達を促す効果的な「足場作り」は次の5つの要素を含むとしている[16]。1) 共同の問題解決：子どもと大人が、楽しみながらお互いに影響を及ぼしあい、ともに目標達成のために励むこと。　2) 相互主観性：お互いに他の人の見方に合わせ、コミュニケーションに関して共通の領域を作り出すこと。　3) 暖かさと応答性：子どもとの共同作業を楽しく暖かく受容的なものにし、また適切に（たとえば“やった！できたね！”というように）ほめたり子どもの能力を認めたりしてあげ、子どもの行動にぴったりと調子を合わせていくこと。　4) 子どもの最近接発達領域をとらえるような関わり：このために、2つのことが必要である。ⅰ）子どもへの要求をいつも達成可能な適切なレベルにあるように課題や子どもを取り巻く環境を調整すること。 ⅱ）大人の介入の度合いをいつも子どもの現在の要求や能力に応じて調整すること。　5) 自己制御の促進：子どもが自分で学習したり自分の行動を統制したりできるようにすることであり、可能な限り大人が手を引いて子どもに責任を持たせること。以上の5要素を親子の遊びのプログラムに取り入れる。「共同の問題解決」のために、本プログラムの遊びを通して親子で共通の目的を達成してもらうようにする。「相互主観性」を育むために、遊びの中で役割を親子で交代してもらい、子どもが相手の立場を考える場を持ち、また、親が相手の立場で考えるところを子どもが見習えるようにする。親の子どもへの「暖かさと応答性」を促進するため、親には、子どものしたことに丁寧に応じできたことをほめて認めてあげることを要請する。「子どもの最近接発達領域を捉えるような関わり」を促すために、子どものできるレベルに対応することができるように遊びの内容にバリエーションを持たせる。「自己制御の促進」のために、遊びのプログラムは子どもが主導して親子で遊んでもらうようにする。まず幼稚園で担任とクラスメートと一緒に遊んで子どもたちに内容を覚えてもらい、それを家で子どもが親に教えて一緒に遊ぶという形式をとる。先行研究では、愛着向上の生活介入プログラムは数か月から半年のように比較的短期間であり、親にとって負担の少ない内容のものが効果的であることがわかっている[26]。このことから、本プログラムの介入期間を3か月間とする。親子の遊びをプログラムとして用いる上で、遊びが成り立たないほど短くなく、また、子どもが飽きたりせずに楽しめるような適切な長さの時間はどのくらいかについて幼稚園教諭との協議の結果、1日10分遊んでもらうこととする。また、認知力向上を目的とした生活介入プログラムでは、1週間の中に適度に休みの日を入れるために、週5日施行することとしているものが多い[4,28]。以上のことから、プログラムは1日10分程度、週5回、3か月間行うものとする。

**7.2.生活介入プログラム変更規準**

参加者が生活介入プログラムを実施することが困難な場合、中止を可とする。

**7.3. 併用療法、支持療法**

特記すべきことなし。

**7.4. 治療中止規準、完了規準**

中止基準：参加者が生活介入プログラムを実施することが困難な場合。

完了基準：参加者が生活介入プログラムを実施できた場合。

**7.5.生活介入プログラム終了後のプログラム実施について**

生活介入プログラムの効果を維持するために行う維持プログラムについては、幼稚園の教育カリキュラムとして継続して行う。

**8　有害事象の評価と報告**

**8.1. 有害事象の定義**

本生活介入プログラムに参加することによって親子やその家族に起こる、有害事象及び重篤な有害事象。

**8.2. 有害事象の評価**

プロトコル実施後に発生した有害事象については、プロトコル実施の特性などを考慮してプロトコル実施終了後30日以内に評価の上、報告する。

**8.3. 予期される有害事象**

本生活介入プログラムは、親子にとって負担の少なく、かつ、楽しめる内容にしてある。また、同種の先行研究からも、予期される有害事象は現段階では認められない。そのため、予期される有害事象は現段階ではない。

**8.4. 有害事象の報告と対応**

もし有害事象を生じた場合は、研究責任者が、研究代表者、東北大学加齢医学研究所所長、東北大学

医学部長、当局などへ、有害事象の情報を得た時点で直ちに報告する。

**9. 検査項目とスケジュール**

介入群・比較群とも、母親に対し、育児ストレスインデックス、子どもの行動チェックリストを、生活加入プログラム実施前（day 0）、実施後 (day 90)、プログラム終了2年後 (day 730) に実施する。

本節で規定された観察・検査項目の結果はすべて報告書に記載され、データとして収集される。

9.1. 観察・検査項目スケジュール

| 評価項目 | プロトコル実施前  (day 0) | プロトコル終了時 (day 90) | フォローアップ時(day 730) |
| --- | --- | --- | --- |
| グッドイナフ人物知能検査　知能指数 | ○ | ○ | ○ |
| 新S-S知能検査　下位項目 | ○ | ○ | ○ |
| 育児ストレスインデックス　子どもの側面 | ○ | ○ | ○ |
| 育児ストレスインデックス　子どもの側面　下位項目 | ○ | ○ | ○ |
| 育児ストレスインデックス　親の側面 | ○ | ○ | ○ |
| 育児ストレスインデックス　親の側面  下位項目 | ○ | ○ | ○ |

**9.2. 登録前・治療開始前の観察・検査項目**

エンドポイント：

こどもの認知発達

主要エンドポイント: グッドイナフ人物画知能検査　知能指数

副次エンドポイント：新S-S知能検査の下位項目

子どものメンタルヘルス

主要エンドポイント：育児ストレスインデックス　「こどもの側面」

副次エンドポイント：育児ストレスインデックス　子どもの側面の下位項目

親のメンタルヘルス

主要エンドポイント：育児ストレスインデックス「親の側面」

副次エンドポイント：育児ストレスインデックス　親の側面の下位項目

**9.3. 生活介入プログラム実施中の観察・検査項目**

なし

**9.4. 治療終了後（追跡期間中）の観察・検査項目**

エンドポイント：

こどもの認知発達

主要エンドポイント: グッドイナフ人物画知能検査　知能指数

副次エンドポイント：新S-S知能検査の下位項目

子どものメンタルヘルス

主要エンドポイント：育児ストレスインデックス　「こどもの側面」

副次エンドポイント：育児ストレスインデックス　子どもの側面の下位項目

親のメンタルヘルス

主要エンドポイント：育児ストレスインデックス「親の側面」

副次エンドポイント：育児ストレスインデックス　親の側面の下位項目

・試験治療が何らかの理由（「7.4. 治療中止規準、完了規準」を参照のこと）で中止又は追跡不能となった場合は、その時期及び理由を調査する。研究分担者は、参加者の権利を十分に尊重した上で、その理由を確認するための適切な努力を払うものとする。

**10. エンドポイント（評価項目）**

**10.1. 有効性エンドポイント**

**10.1.1. 主要エンドポイント**

こどもの認知発達：グッドイナフ人物画知能検査　知能指数

子どものメンタルヘルス：育児ストレスインデックス　「こどもの側面」

親のメンタルヘルス：育児ストレスインデックス「親の側面」

**10.1.2. 副次エンドポイント**

こどもの認知発達：新S-S知能検査の下位項目

子どものメンタルヘルス：育児ストレスインデックス　子どもの側面の下位項目

親のメンタルヘルス：育児ストレスインデックス　親の側面の下位項目

**11. 統計学的事項**

**11.1. 解析対象集団**

介入群、比較群

**11.2. 有効性の主要評価項目の解析**

本介入プログラムの実施前、実施後の差について、両側t検定にて効果を評価する。

**11.3. 有効性の副次的評価項目の解析**

本介入プログラムの実施前、実施後の差について、両側t検定にて効果を評価する。

**11.4. サンプルサイズ、予定登録期間、追跡期間**

対象はわかくさ幼稚園の年中組・年中組の児童とその母親。調査に同意しない母子が一部いることを見込み、およそ200～220組が本調査に参加すると予想する。

予定登録期間：5年間

追跡期間：2年間

**12. 倫理的事項**

**12.1. 患者の保護**

本試験はヘルシンキ宣言（2002年改訂）に基づく倫理的原則を遵守し、改正GCP（2003年改正）及び臨床研究に関する倫理指針の改正等について（医政発第0731002号）を準用して実施する。

**12.2. 患者への説明と同意（インフォームド・コンセント）**

試験への登録に先立ち、研究代表者は、患者が本試験に参加する前に、東北大学医学部第2倫理委員会及び各施設のInstitutional Review Board (IRB)で承認の得られた同意説明文書を用いて、研究参加者本人に十分に説明し、本試験への参加について自由意志による同意を文書により得る。

同意書には説明を行った参加者が記名捺印又は署名し、各自日付を記入する。研究分担者は、参加者

が本試験に参加する前に、説明文書を参加者に渡し、口頭及び文書で説明する。記名捺印または署名

された同意書は東北大学加齢医学研究所スマートエイジング国際共同研究センターで施錠管理する。

同意書は5年間保管される。

本研究には未成年者とその親を対象とするが、未成年者への配慮として、下記のようなことを行う。

保護者説明会で説明した内容（生活介入プログラム内容、参加するか否かは自由に選択できること、またいつでも参加を取りやめることができること、調査への不参加や途中で参加を取りやめることは幼稚園における園児の評価に影響を与えないこと）を保護者から家庭で子供に説明してもらう。また、幼稚園でもクラスで担任から同じことを子供に説明してもらう。また、保護者への説明会当日、調査者から子供へ同内容（生活介入プログラム内容、参加するか否かは自由に選択できること、またいつでも参加を取りやめることができること、調査への不参加や途中で参加を取りやめることは幼稚園における園児の評価に影響を与えないこと）の説明を口頭で行う。本人の同意については、保護者に代理で記入してもらう。

**12.3. プライバシーの保護**

個人情報は、連結可能匿名化し、連結表を東北大学加齢医学研究所スマートエイジング国際共同研究センター内で施錠管理する。

**12.4. 実施計画書の遵守**

本試験に参加する研究者は、患者の安全と人権を損なわない限り、本実施計画書を遵守する。

**12.5. 東北大学第２倫理委員会による承認**

本試験実施前及び試験実施予定期間中を通じて、東北大学医学部第2倫理委員会において、本試験の実施、継続等について倫理的、科学的及び医学的妥当性の観点から承認を得る。研究代表者は、実施計画書、説明同意文書など審査の対象となる文書を東北大学医学部第2倫理委員会に提出する。

**12.6. 新たな情報の報告**

本試験に用いる全ての内容の有効性、安全性に関する新たな情報を得た場合、研究代表者は必要に応じて、わかくさ幼稚園に報告する。白菊幼稚園教諭はその旨を通知し、参加者に対する適切な生活介入プログラムの実施及び事後処理を保証する。

**12.7. プロトコルの内容変更について**

プロトコルの内容を変更する際には、「15. プロトコルの内容変更」に従い、効果・安全性評価委員会に改訂の申請を行い、承認を得る。倫理審査委員会に審査承認を要するかどうかは東北大学加齢医学研究所スマートエイジング国際共同研究センターの取り決めによる。

**13. 費用負担と補償**

**13.1. 資金源及び財政上の関係**

東北大学21世紀COEプログラム「言語・認知総合科学戦略研究拠点」

**13.2. 試験にかかる費用負担**

試験期間中の費用の負担はなし。

**13.3. 健康被害の補償及び保険への加入**

**13.3.1. 健康被害の補償**

対象者に健康被害を生じる可能性は極めて低いため、本試験では補償はない。

**13.3.2. 補償・賠償保険への加入**

なし

**14. モニタリングと監査**

**14.1. モニタリング**

子どもが親に家で遊びを教える形をとるが、正確に遊びの内容が親に伝わるように、１か月に1枚、4種類の遊びについて記載したプリントを配布する。そのプリントには4種類の遊びに4色の色を対応させる。それぞれの色について5枚ずつのシールとその月のカレンダーを各家庭に配布し、親子で遊んだ後に子どもがその遊びに対応する色のシールを、カレンダーのシートに張ってもらうこととして、取り組む遊びの種類が偏らず、均等に遊べるようにする。尚、何の遊びをどの日に行うかについては、各家庭で子どもに任せることとする。カレンダーは回収しないが、このシールを張るシステムにより、参加者のプログラム施行へのモチベーションを高める。

生活介入プログラム実施後のアンケート

比較群の介入終了後の4週間後に、介入期間中の脳トレ遊びの頻度についてアンケートを実施する。「脳トレ遊びは、毎日実施されましたか？」という質問に対し、

1. 毎日　2．2日に1回程度　3．週に2回程度　4．週に1回程度

5．10日に1回程度

の5択の選択肢の中から回答を要請する。本研究のようなクロスオーバーデザインでは、介入群と比較群で参加者のモチベーションが異なり、後半に生活介入を実施する群でモチベーションが下がることがある[29]。そのため、アンケート解析は、介入群のみを対象とすることとする。

**14.2. プロトコル違反・逸脱**

モニタリングによりプロトコル違反・逸脱が発見された場合の対処法を記載する。

違反 Violation

プログラム中に、幼稚園側に参加者から何らかの理由でプログラムを行えないなど、本研究への参加の辞退の申し出があった場合は、それを認める。

許容範囲 Acceptable deviation

参加者がプログラムを行えていないと何らかの理由で判明した場合も、参加者の方から本研究への参加の辞退の申し出がなければ、参加を継続してもらうこととする。

**15. プロトコルの内容変更**

プロトコルの改正又は改訂の手続き方法について記載する。

実施計画書の内容を変更する場合には、変更に先立ち、「プロトコルの内容変更申請書」を効果・安全性評価委員会に提出し、承認を得る。

変更内容が試験実施計画の重要な変更と考えられる否かによって、以下の改正か改訂に相当するかは効果・安全性評価委員会が決定し承認する。

改正 Amendment

試験の被験者のリスクを増大させる、試験の主要評価項目に関係するなどの試験計画の重要な変更と考えられる変更。東北大学医学部第2倫理委員会の承認を得る。

改訂 Revision

試験の被験者のリスクを増大させる可能性がない、試験の主要評価項目に関係しないなどの試験計画の軽微な変更。重大性に関わらず、全ての改定内容とその理由を東北大学医学部倫理審査委員会に報告する。

- 改定内容が重大と判断される場合、東北大学医学部倫理審査委員会での再審査および承認を得る。
- 重大と判断されるプロトコルの改正とは、以下のいずれかの項目が変更されることをいう。

1. 試験デザイン
2. 研究対象（適格基準）
3. エンドポイント
4. 目標症例数
5. 予期される有害事象

- プロトコルに改定があった場合には、研究代表者は、それに応じて被験者への説明文書を改定する。

**16. 試験の終了と早期中止**

本生活介入プログラムによって、介入群に著しい有害事象が生じることが判明した場合、試験を早期中止するものとする。

**17. 記録の保存**

収集したデータについては研究代表者が5年間保管するものとする。原資料（診療記録等）ならびに試験実施機関で保管される書類（実施計画書、同意説明文書等）については、2年間保持するものとする。

**18. 研究結果の帰属と発表**

試験で得られた研究成果の帰属と研究結果を公表（学会発表・論文投稿など）する場合の手続きについて記載する。

・ICMJEの勧告に基づき、UMIN臨床試験登録に登録する。

・試験で得られた研究成果は東北大学加齢医学研究所スマートエイジング国際共同研究センターに帰属するものとする。本研究の発表の際の著者順位は、立花良之、福島愛、斉藤仁美、米山哲史、牛田和夫、米山進、川島隆太の順とし、corresponding authorは立花良之とする。

**19. 研究組織**

研究代表者 川島隆太

主任研究者　立花良之

研究分担者　福島愛、斉藤仁美、米山哲史、牛田和夫、米山進

研究事務局　東北大学加齢医学研究所スマートエイジング国際共同研究センター

　　　　　　　　〒980-8575 宮城県仙台市青葉区星陵町4‐1

　　　　　　　　TEL 022-717-7988

参加施設　わかくさ幼稚園

　　　　　　　〒981-0901 宮城県仙台市青葉区北根黒松16－1

TEL 022-234-5587

**20. 文献**

1. Tsujimoto S (2008) The prefrontal cortex: functional neural development during early childhood. Neuroscientist 14: 345-358.

2. Ito M (2004) ‘Nurturing the brain’as an emerging research field involving child neurology. Brain and Development 26: 429-433.

3. Roland PE, Eriksson L, Stone-Elander S, Widen L (1987) Does mental activity change the oxidative metabolism of the brain? Journal of Neuroscience 7: 2373-2389.

4. Kawashima R, Okita K, Yamazaki R, Tajima N, Yoshida H, et al. (2005) Reading aloud and arithmetic calculation improve frontal function of people with dementia. J Gerontol A Biol Sci Med Sci 60: 380-384.

5. Uchida S, Kawashima R (2008) Reading and solving arithmetic problems improves cognitive functions of normal aged people: a randomized controlled study. Age 30: 21-29.

6. Elliott R (2003) Executive functions and their disorders. Br Med Bull 65: 49-59.

7. Garon N, Bryson SE, Smith IM (2008) Executive function in preschoolers: a review using an integrative framework. Psychol Bull 134: 31-60.

8. Greenberg MT (2006) Promoting resilience in children and youth: preventive interventions and their interface with neuroscience. Ann N Y Acad Sci 1094: 139-150.

9. Blair C, Diamond A (2008) Biological processes in prevention and intervention: the promotion of self-regulation as a means of preventing school failure. Dev Psychopathol 20: 899-911.

10. Thomas KM, King SW, Franzen PL, Welsh TF, Berkowitz AL, et al. (1999) A developmental functional MRI study of spatial working memory. Neuroimage 10: 327-338.

11. Kane MJ, Engle RW (2002) The role of prefrontal cortex in working-memory capacity, executive attention, and general fluid intelligence: An individual-differences perspective. Psychonomic Bulletin & Review 9: 637-671.

12. Vygotsky LS (1978) Mind in society.

13. Wood D, Bruner JS, Ross G (1976) The role of tutoring in problem solving. J Child Psychol Psychiatry 17: 89-100.

14. Jernberg AM, Booth PB (1999) Theraplay: Helping Parents and Children Theraplay: Helping Parents and Children Build Better Relationship through Attachment-based Play Build Better Relationship through Attachment-based Play. San Francisco: Jossey-Bass Publishers.

15. Jernberg AM, Booth PB, Koller TJ, Allert A (1982) Preschoolers and School Age Children in Interaction with Their Parents: Manual for Using the Marschak Interaction Method (MIM). Chicago, IL: Theraplay Institute.

16. Berk LE, Winsler A (1995) Scaffolding children's learning: Vygotsky and early childhood education: National Association for the Education of Young Children Washington, DC.

17. van IMH, Juffer F, Duyvesteyn MG (1995) Breaking the intergenerational cycle of insecure attachment: a review of the effects of attachment-based interventions on maternal sensitivity and infant security. J Child Psychol Psychiatry 36: 225-248.

18. Bowlby J (1977) Maternal care and mental health: Jason Aronson.

19. Bowlby J (1980) Attachment and loss: Basic Books.

20. Bowlby J (1988) A secure base: Clinical applications of attachment theory: Routledge.

21. Bowlby J (1979) The Making and Breaking of Affectional Bonds. London. Tavistock Publications.

22. Howes C, Smith EW (1995) Children and their child care caregivers: Profiles of relationships. Social Development 4: 44-61.

23. Sroufe LA (2005) Attachment and development: a prospective, longitudinal study from birth to adulthood. Attach Hum Dev 7: 349-367.

24. Marvin R, Cooper G, Hoffman K, Powell B (2002) The Circle of Security project: Attachment-based intervention with caregiver-pre-school child dyads. Attachment & Human Development 4: 107-124.

25. Denham SA, Burton R (1996) A social-emotional intervention for at-risk 4-year-olds. Journal of School Psychology 34: 225-245.

26. Bakermans-Kranenburg MJ, Van Ijzendoorn MH, Juffer F (2003) Less is more: meta-analyses of sensitivity and attachment interventions in early childhood. Psychological Bulletin 129: 195.

27. Taylor TK, Biglan A (1998) Behavioral family interventions for improving child-rearing: A review of the literature for clinicians and policy makers. Clinical child and family psychology review 1: 41-60.

28. Klingberg T, Forssberg H, Westerberg H (2002) Training of working memory in children with ADHD. J Clin Exp Neuropsychol 24: 781-791.

29. Craik FIM, Winocur G, Palmer H, Binns MA, Edwards M, et al. (2006) Cognitive rehabilitation in the elderly: Effects on memory. Journal of the International Neuropsychological Society 13: 132-142.

**21. 付録**

**脳を育む遊びによる親子の愛着形成及び認知力向上の検証**

**調査実施についての説明書**

平成20年9月　日

　　わかくさ幼稚園　理事長　米山進

東北大学加齢医学研究所　教授　川島隆太

**はじめに**

　この説明は、保護者の方があらかじめ、この調査のことを正しく理解した上で、自由な意志に基づいてこの調査に参加していただくかどうか判断していただくために行います。

**１．この調査の目的**

東北大学加齢医学研究所とわかくさ幼稚園が共同で行う、前頭葉機能を取り入れた遊びを家庭で実施する調査企画です。「『脳を育む遊び』を毎日楽しむ」という親子の共同作業が、親子関係や認知発達にどのような影響を及ぼすかについて調べます。

**２．調査の方法について**

別紙　資料のような形で行わせていただきます。

**３．参加に同意した場合でも、随時これを撤回できることについて**

この調査を受けるかどうかはお子様および保護者の方の自由です。この調査を受けることを同意した後でも、やめたくなった場合には、いつでも辞退することができます。その場合には、保護者の方か、保護者の方から請託を受けた代理人の方が、「同意を取り消す旨の書面」を下記宛にお送りください。

送付先：　〒980-8579　仙台市青葉区星陵町４－１

　　　　　東北大学加齢医学研究所脳機能開発研究分野

　　　　　川島隆太　宛

　　　　　電話022-717-7988（担当：立花良之）

**５．調査にかかる費用について**

　この調査には一切費用はかかりません。

**６．その他、参加者のプライバシーの保護に関し必要な事項について**

　得られたデータは学術目的にのみ使用されます。この調査に関することでお子様や保護者の方の名前が出ることはなくプライバシーは厳重に守られます。調査終了後も同じです。この調査について詳しく説明をしてもらいたいことや、心配なことがあれば、いつでも遠慮なくお申し出下さい。

　このプロジェクトでご提出いただいた同意書は、東北大学加齢医学研究所に調査終了まで厳重に施錠保管いたします。管理責任者は、東北大学加齢医学研究所教授川島隆太です。なお、承諾の取り消しがあり次第、承諾書は焼却処分します。

**７．実施対象**

わかくさ幼稚園に通園中のお子様及びその保護者の方

**８．研究組織**

　この調査は、主に次の研究者らの協力と指導のもとに行われます。

研究代表者

・東北大学　加齢医学研究所教授　医学博士　川島隆太

研究分担者

・東北大学加齢医学研究所脳機能開発研究分野

　　　　　　　　　　　　　福島愛

　　　　　　　　　　　　　立花良之(児童精神科医)

　わかくさ幼稚園　　　　　斉藤仁美、

米山哲史、

牛田和夫、

米山進

　以上の説明を十分に理解されてこの調査にご参加いただけるようでしたら、別紙の同意書に署名、捺印をお願いします。

（介入群　保護者用資料）

**脳を育む遊びによる親子の愛着形成及び認知力向上の検証**

**調査ご協力のお願い**

【研究の目的】「毎日楽しんで親子で『脳を育む遊び』を行う」という体験が、親子関係やお子さんの発達にどんな影響を及ぼすかについて調べます。

【お願いさせていただきたいこと】

1. 9月8/9/10日に、本調査についての説明会を行わせていただきます。
2. 毎日10分脳を育む遊びを親子で実行してください。意識的に遊びの時間に、お子さんができたことをほめて認めてあげください。
3. 遊びシートにその日行った遊びに対応するシールを、お子さんに貼ってもらってください。
4. 9/8の週、12/8の週、3/8の週におうちやお子さんのことについてのアンケートに答えていただきます。

【全体の流れ】

**前半3ヶ月**

**後半3ヶ月**

**親子で毎日**

**脳を育む遊びを実施**

**1回目**

**アンケート**

**2回目**

**アンケート**

9/8～

12/8～

3/8

**脳を育む遊び実施なし**

アンケートにつきましては、お忙しいところ大変恐縮ですが、1週間以内にご回答いただき幼稚園の先生にお渡しください。

(B群　保護者用資料)

**脳を育む遊びによる親子の愛着形成及び認知力向上の検証**

**調査ご協力のお願い**

【研究の目的】「毎日楽しんで親子で『脳を育む遊び』を行う」という体験が、親子関係やお子さんの発達にどんな影響を及ぼすかについて調べます。

【お願いさせていただきたいこと】

1. ９月8 /9 /10日に、本調査についての説明会を行わせていただきます。
2. 後日（12月）に親子での脳を育む遊びについての説明会を行わせていただきます。
3. 9/8の週、12/8の週、3/8 の週におうちやお子さんのことについてのアンケートに答えていただきます。

【全体の流れ】

**前半3ヶ月**

**後半3ヶ月**

**親子で毎日**

**脳を育む遊びを実施**

**※説明会**

**2回目**

**※後半期間開始時に、「脳を育む遊び」の生活介入について説明会を行わせていただきます。**

**脳を育む遊び実施なし**

9/8～

12/8～

3/8

**アンケート**

**アンケート**

**1回目**

アンケートにつきましては、お忙しいところ大変恐縮ですが、各回1週間以内にご回答いただき幼稚園の先生にお渡しください。

(介入群　園児用資料)

**同意書**

この度、「脳を育む遊びによる親子の愛着形成及び認知力向上の検証」の調査の目的、方法、予想される効果および安全性等について、担当者より十分な説明を受けましたので、調査の実施を承諾いたします。

平成　　年　　月　　日

東北大学　加齢医学研究所　所長

　　　　　　　　　　　　　　　　　福　田　　　寛　殿

東北大学　加齢医学研究所　教授

　　　　　　　　　　　　　　　　　川　島　隆　太　殿

わかくさ幼稚園　理事長

　　　　　　　　　　　　　　　　　米　山　進　殿

保護者署名　　　　　　　　　　　　　　　印

住所　（上記と異なる場合お書きください）

**なにかご不明な点がございましたら**

**〒980-8575　仙台市青葉区星陵町４－１**

**東北大学加齢医学研究所脳機能開発研究分野**

**TEL　022-717-8468**

**担当　立花良之　にお問い合わせください。**

**「脳を育む遊びによる親子の愛着形成及び認知力向上の検証」**

**調査参加についての同意の撤回**

私 は、「脳を育む遊びによる親子の愛着形成及び認知力向上の検証」（代表者　川島隆太）に協力することを同意しましたが、この同意を撤回いたします。

平成　　年　　月　　日

同意者氏名： 印

同意者住所：

（代理人の場合）

代理人氏名： 印

代理人住所：

**同意の撤回をされる時には、この用紙を**

**〒980-8575　仙台市青葉区星陵町４－１**

**東北大学加齢医学研究所脳機能開発研究分野**

**川島隆太　宛にお送りください。**

**また、何かご不明な点がございましたら、**

**TEL　022-717-8468　脳機能開発研究分野　担当　立花良之**

**までお問い合わせください。**

※注

11.2、11.3：　PLoS ONE の査読者の助言により、投稿後、解析手法を混合モデルに変更した。
