# Supplementary material for: A New Mother-Child Play Activity Program to Decrease Parenting Stress and Improve Child Cognitive Abilities: A Cluster Randomized Controlled Trial
Source: PLoS One. 2012 Jul 27;7(7):e38238. doi: 10.1371/journal.pone.0038238 (PMC3407189; doi:10.1371/journal.pone.0038238)
Supplement: Appendix S1 — The details of the program. (PDF) [file pone.0038238.s003.pdf]

Appendix S1. The details of the program

Name of the play activity	Instruments used in the play activity	Target of cognitive functions	Referenced neurological tasks	Brain area associated with neuropsychological tasks	Contents of the play activity	Advanced play activities	References
Freezing	Something which releases sounds; musical instruments (tambourine, castanet, etc.)<br>CD<br>Bell<br>etc.	Inhibition	Go/No-go	Dorsolateral prefrontal cortex	1. Children and their mothers dance joyfully to the music.<br>2. Children explain the rules to their mothers. The rules are that their mothers should freeze when the sounds stop.<br>3. Children and their mothers enjoy the play activities by switching roles.<br>4. If children succeed at freezing, their mothers praise them.	1. Mothers bump up the time of freezing.<br>2. Mothers reverse the rules. Children dance when the sound isn't heard and freeze when it is heard.<br>3. Multiple musical instruments and rules can be used (e.g. children dance when the tambourine sounds but freeze when the castanet sounds).	Banich et al., 2000<br>Konishi et al., 1999
Motion	Three types of illustrated cards<br>・ animal<br>・ vehicle<br>・ food	Decision-making<br>Inhibition	Decision-making	Dorsolateral prefrontal cortex	1. Children explain to their mothers the names and motions of the animals illustrated on the cards.<br>2. When children show the cards to their mothers, mothers act out the animals' motions.<br>3. When children clear all the play activities with the animal cards, the activities are stopped.	1. Mothers increase the speed gradually.<br>2. When children finish the play activities with the animal cards, children try the play activities with the vehicle cards and the food cards. When mothers show the vehicle cards, children walk around their mothers. When mothers show the food cards, children crouch down.<br>3. When children adjust to the play activities with the vehicle cards and the food cards, mothers perform the play activities with the three card types mixed together.	Rourie et al., 2005

Inhibition	None	Inhibition abilities<br>Controlling behaviors or complex operation	Go/No-go	Dorsolateral prefrontal cortex	1. Children and their mothers clap their hands while singing songs (the songs are children's songs which they often sing while clapping their hands).<br>2. Children don't clap their hands at the parts when they usually would.<br>3. Children change the phrases at some parts of the song.<br>4. Children and their mothers switch roles in turn.	1. Mothers change the speed of the songs.<br>2. Mothers can change to other songs.	Aron et al., 2005
Imitation	None	Communication abilities	Imitation	Inferior frontal gyrus	1. Children ask their mothers to mimic their facial expressions.<br>2. Mothers try to imitate their children's emotions from their facial expressions.<br>3. Children and their mothers switch roles in turn and mimic each other.	Mothers ask their children to mimic their postures and facial expressions in more complicated ways.	Leslie et al., 2005
Categorization	White paper<br>Pen	Categorization<br>Memory<br>Evoking<br>Language	Categorization	Dorsolateral prefrontal cortex	1. Children choose themes, and imagine objects that match those themes. (e.g. for the theme “something round” children might imagine a ball.)<br>2. Children draw the objects they imagined on paper.<br>3. Children and their mothers talk about the themes and the pictures.<br>4. Mothers should not disapprove of whatever their children might draw, but should praise them for all their answers.<br>5. Mothers and children repeat steps 1 through 4 until the paper is full of pictures.	Mothers draw pictures and show them to their children. The children find the mutual characteristics among them and categorize them.	Lau et al., 2007<br>Aron et al., 2005
Gaze detection	None	Considering others<br>Communication	Mentalizing	Medial prefrontal cortex<br><br>Orbitofrontal cortex	1. Children look at an object in the room and ask their mothers what they are looking at.<br>2. Mothers make guesses at what their children are looking at.<br>3. Children and their mothers switch roles in turn.	Mothers change the targets from nearby, easy objects to distant, difficult objects.	Kroger et al., 2002

Tongue twister	White paper<br>Pen	Manipulating words	Language	Inferior frontal gyrus	1. Children and their mothers play tongue twisters.<br>2. Mothers praise their children when they succeed.<br>3. Mothers show cards on which tongue twisters are written. Children select some cards and try the tongue twisters.<br>4．Children and mothers do tongue twisters with each other.		Fooker et al., 2008
Silent touch	None	Communication<br>Inhibition<br>Concentration	Inhibition<br>Attention	Dorsolateral prefrontal cortex	1. Children move their mouths as if they were saying words, but do not use their voices.<br>2. Mothers pay attention to their children's mouths and infer what their children are saying.<br>3. Children give the answer with gestures and not voices.<br>4. Children tell their mothers the answers.<br>5. Children and their mothers switch roles in turn.<br>6. If children' s gestures are correct, their mothers praise them.		George et al., 2007<br>Aron et al., 2005
Counting	A paper with the numbers written on it	Numerical cognition	Arithmetic	Dorsolateral prefrontal cortex	1. Children and their mothers count things in their house in turn.<br>2. Mothers count the numbers written on a piece of paper while pointing.<br>3. Children and their mothers count the numbers with one another.	1. After children become accustomed to the previous activities, mothers count the numbers on the paper in reverse.<br>2. Next, mothers increase the numbers by sets of ten until the activity is sufficiently challenging for the child.	Kroger et al., 2002
Inferring others' intentions	Bucket<br>Towels<br>Garbage box<br>etc.	Considering others<br>Prediction	Mentalizing	Medial prefrontal cortex<br><br>Orbitofrontal cortex	1. Children ask their mothers to infer their' intentions.<br>2. Children perform activities from daily life (e.g. if children want to express “cleaning”, they use household objects and make gestures as if they were cleaning).<br>3. Mothers make guesses at their children's intentions.<br>4. Mothers and children confirm the answers to the children's intentions.<br>5. Children and their mothers switch roles in turn.		Fooker et al., 2008

Mimicking animal sounds	Cards of illustrated animals	Communication	Imitation	Inferior frontal gyrus (IFG)	1. Children show illustrated animal cards to their mothers and ask them to say the animals' names.<br>2. Children ask their mothers to make the animals' sound.<br>3. Then children ask their mothers how the same animal sounds under other conditions (for example, "when they are sad", "when they are glad" etc.).<br>4. Children and their mothers switch roles in turn.		Iacoboni, 2005
Going on errands	Illustrated cards<br>or trump Cards	Categorization<br>Memory<br>Decision-making	Categorization<br>Working memory<br>Decision-making	Dorsolateral prefrontal cortex	1.Children arrange the cards while saying the pictures' names and categories.<br>2. Children ask their mothers to go on errands. (e.g. "go to Mr. Dog's shop and buy tomatoes.")<br>3. Mothers pick up the cards as their children give them orders.<br>4. Children and their mothers switch roles in turn.<br>5. At the end of the game, children gather the cards and put them into the box according to their categories.	1. Mothers make their requests more complicated by adding other card categories. (e.g. "take a car to Mr. Dog's shop and buy tomatoes.")<br>2. Mothers change the categories of the cards according to the other characteristics of the objects on the cards and children pick of the cards of the named category. (e.g. "the number of characters in the name" or "colors" etc.)<br>(If children pick up an unexpected but correct card, mothers praise them).	Barch et al., 1997<br>Vogels et al., 2002
Picking up Objects	Chopsticks<br>Dishes<br>Small objects (beads, marbles, colored macaroni etc.)	Decision-making	Decision-making	Dorsolateral prefrontal cortex	(Children who can't use chopsticks pick up objects with their hands.)<br>1. Children set objects for picking up on dishes.<br>2. Children decide the numbers and colors of the objects that they want picked up and ask their mothers to pick up the objects according to their instructions.<br>3. Mothers and children take turns giving instructions and picking up the objects.	1. Adding other family members to the play activities makes it more exciting.<br>2. Mothers add other varieties of objects to the activity and also change the instructions.	Ridderinkhof et al., 2004

Differences	Illustrated sets of cards which are similar but have slight differences	Discrimination<br>Working memory	Discrimination<br>Working memory	Dorsolateral prefrontal cortex	1. Children show their mothers sets of pictures which are similar but have some differences.<br>2. Children ask their mothers to clarify the differences between the two pictures.<br>3. Children and their mothers switch roles in turn.	1. After children become accustomed to the plays, their mothers show sets of belongings (food, plates etc.) and children clarify the differences between them.<br>2. Mothers ask their children about both the similarities the differences in the objects.<br>3. After children become accustomed to the play activities, children are told to remember the objects and close their eyes or turn their backs. Children then name the object's similarities and differences from memory.	Awh et al., 2006
Rock-paper<br>-scissors	None	Inhibition<br>Communication	Inhibition	Dorsolateral prefrontal cortex	1. Children and their mothers play rock-paper-scissors.<br>2. In the play activities, when mothers and children make the gestures, they will say the names.<br>(e.g. when children show scissors, they say "scissors").<br>3. In the next step, mothers are asked to allow themselves to be defeated (for example, immediately after children say "rock", mothers show "scissors").<br>4. Children and their mothers switch roles in turn.	1. After children become accustomed to the play activities, their mothers increase the speed.<br>2. In mid-course of the play activities, mothers and children return to playing ordinary "rock-paper-scissors".	Matsuda et al.,<br>2004
Maze game	Pen<br>Maze activity paper	Spatial cognition<br>Decision-making	Decision-making	Dorsolateral prefrontal cortex	Children play the maze games with a pen.	1. After children become accustomed to the play activities, they solve the maze puzzles within a time limit.<br>2. Mothers adjust the difficulty of the maze puzzles according to their child's level.	Owen, 1997

Word fluency	Paper<br>Pen<br>Letter chart	Communication abilities<br>Word manipulation<br>Imaging abilities	Word fluency	Inferior frontal gyrus	1. Children are told to generate a word with its initial syllables assigned (i.e. /a/ka/sa/ta/na/etc.).<br>2. Mothers write down or draw a picture about the words which their children generate for the assigned syllable in a 3×3 table on paper.<br>3. When the table is filled with words or pictures, children and their mothers say the words aloud.<br>4. After finishing the exercise, mothers praise their children for their good efforts with physical affection.		Kane et al., 2002
Memory	Illustrated cards or<br>trump cards	Working memory	Working memory	Dorsolateral prefrontal cortex	1. Children set down cards with their illustrated sides up.<br>2. Their mothers remember the cards in their minds.<br>4. Children turn the cards face-down.<br>5. Mothers make guesses at which cards are which.<br>6. Children and their mothers switch roles in turn.<br>7. When children's answers are correct, their mothers praise them.	1. The numbers of cards can be increased.<br>2. After children have memorized all the cards, mothers insert another play activity to make it more difficult for children to remember and identify the cards later. (e.g. singing a song with the children.)	Thomas et al., 1999
