# Supplementary material for: A New Mother-Child Play Activity Program to Decrease Parenting Stress and Improve Child Cognitive Abilities: A Cluster Randomized Controlled Trial
Source: PLoS One. 2012 Jul 27;7(7):e38238. doi: 10.1371/journal.pone.0038238 (PMC3407189; doi:10.1371/journal.pone.0038238)
Supplement: Appendix S2 — The Parenting Stress Index mean score change in each class in the intervention and control groups. (PDF) [file pone.0038238.s004.pdf]

Appendix S2. The Parenting Stress Index mean score change in each class in the intervention and control groups

	class name	C1	C2	C3	C4	I1	I2	I3	I4	I5
Child domain	Reinforces	1.11	-0.85	-0.89	0.43	0.04	0.00	0.42	-1.65	0.11
	parent	(3.46)	(2.54)	(4.59)	(2.24)	(3.10)	(2.02)	(2.26)	(5.49)	(1.97)
	Mood	0.68	-0.73	-0.56	-0.03	-0.35	-1.71	-2.29	-0.18	-0.85
		(3.62)	(2.46)	(6.33)	(2.96)	(2.06)	(3.05)	(4.13)	(2.56)	(3.99)
	Demandingness	0.89	-0.69	-0.94	0.07	-0.65	-1.14	-0.13	-0.94	-0.48
		(2.51)	(2.33)	(3.49)	(2.05)	(2.79)	(2.06)	(2.68)	(2.97)	(1.91)
	Distractibility	0.29	-0.88	-0.33	-0.83	-1.04	-0.52	-1.13	0.12	-0.52
	/Hyperactivity	(2.66)	(2.50)	(4.07)	(2.82)	(2.62)	(2.93)	(2.23)	(2.47)	(2.10)
	Adaptability to people	0.32	-0.58	-0.89	0.10	-0.52	-2.00	-0.50	-1.71	-0.44
		(2.92)	(2.27)	(4.27)	(2.47)	(2.25)	(3.07)	(2.95)	(2.39)	(1.93)
	Acceptability to parent	0.21	-0.58	0.11	-0.09	-0.87	-0.62	-0.21	-0.18	-0.92
		(2.27)	(2.12)	(3.50)	(2.58)	(1.71)	(2.42)	(2.26)	(2.32)	(3.05)
	Adaptability to things	0.50	-0.08	-0.67	-0.04	-0.91	-0.57	-0.83	-0.76	-0.07
		(2.08)	(1.87)	(2.79)	(2.03)	(2.00)	(2.68)	(1.81)	(2.49)	(1.52)
	Total score	4.00	-4.38	-4.17	1.55	-4.17	-6.57	-4.67	-5.29	-4.17
	of child domain	(13.13)	(8.86)	(21.99)	(10.58)	(8.62)	(10.59)	(11.50)	(14.68)	(9.29)
Parent domain	Role restriction	0.86	-1.08	0.94	-0.73	0.35	0.29	0.96	0.41	-0.78
		(4.27)	(3.71)	(5.94)	(3.91)	(3.65)	(4.01)	(3.61)	(3.69)	(2.68)
	Isolation	1.43	0.50	0.33	-0.10	0.00	0.00	0.21	0.35	-0.82
		(2.97)	(2.27)	(6.15)	(3.16)	(3.21)	(3.27)	(2.75)	(3.24)	(2.64)
	Spouse	0.14	0.04	1.17	-0.30	-0.65	-0.14	-0.08	0.65	0.11
		(2.26)	(2.88)	(5.18)	(2.76)	(2.74)	(2.71)	(3.05)	(2.83)	(4.17)
	Competence	0.05	-0.54	0.17	0.09	-0.22	-0.71	-0.92	-2.06	-0.50
		(2.67)	(2.10)	(3.59)	(2.48)	(2.76)	(2.97)	(2.21)	(2.22)	(2.14)
	Depression	-0.50	-0.35	-0.78	0.50	-0.09	-0.43	-0.21	-1.41	-0.56
		(2.99)	(2.45)	(3.87)	(2.29)	(1.83)	(2.20)	(2.30)	(3.16)	(2.72)
	Attachment	0.18	-0.62	-1.06	0.23	-0.43	0.19	-0.21	-0.88	0.00
		(1.85)	(1.90)	(2.90)	(1.94)	(2.11)	(1.83)	(1.41)	(2.09)	(2.18)
	Health	-0.18	0.12	0.50	0.20	-0.22	-0.57	-0.58	-0.35	0.00
		(1.91)	(2.32)	(3.31)	(1.99)	(2.04)	(2.40)	(2.21)	(1.46)	(2.60)
	Total score	2.20	-1.92	1.67	1.29	-1.30	-1.48	-0.79	-4.00	-3.33
	of parent domain	(11.03)	(10.43)	(23.26)	(9.42)	(11.27)	(11.27)	(11.48)	(12.76)	(10.53)

See Table 1 legend. ( ) indicates the standard deviation of the mean change.
