# Supplementary material for: A New Mother-Child Play Activity Program to Decrease Parenting Stress and Improve Child Cognitive Abilities: A Cluster Randomized Controlled Trial
Source: PLoS One. 2012 Jul 27;7(7):e38238. doi: 10.1371/journal.pone.0038238 (PMC3407189; doi:10.1371/journal.pone.0038238)
Supplement: Appendix S3 — The Goodenough Draw-a-man Intelligence Test and the New S-S Intelligence Test mean score change in each class in the intervention and control groups. (PDF) [file pone.0038238.s005.pdf]

Appendix S3. The Goodenough Draw-a-man Intelligence Test and the New S-S Intelligence Test mean score change in each class in the intervention and control groups

	subscale	C1	C2	C3	C4	I1	I2	I3	I4	I5
The DAM	Intelligence Quotient	2.29 (14.23)	0.38 (10.79)	1.39 (11.36)	-0.01 (14.39)	2.65 (17.23)	4.23 (13.37)	7.56 (17.01)	5.07 (16.65)	5.58 (16.08)
The NS-SIT	Understanding relationships between things	2.31 (2.84)	2.12 (2.37)	1.62 (3.19)	3.03 (2.48)	3.58 (3.32)	1.63 (2.41)	2.89 (1.95)	3.17 (2.93)	3.33 (3.31)
	Counting and comparing the numbers	1.19 (1.92)	0.36 (1.44)	0.71 (2.10)	0.95 (1.75)	0.63 (1.54)	0.58 (1.92)	0.70 (1.27)	1.09 (1.59)	0.82 (1.87)
	Calculation	1.08 (1.47)	1.36 (1.58)	0.19 (1.50)	0.45 (1.15)	1.16 (1.38)	0.95 (1.39)	0.78 (1.22)	0.65 (1.72)	0.55 (1.10)
	Completion of the pictures	2.85 (1.49)	3.08 (1.80)	0.76 (2.07)	1.21 (1.67)	2.74 (2.33)	3.42 (2.36)	3.26 (1.91)	1.17 (1.23)	1.31 (1.67)
	Working memory	0.12 (1.66)	0.76 (1.59)	0.38 (1.99)	-0.23 (1.59)	0.84 (1.46)	0.58 (1.61)	0.70 (1.75)	0.35 (1.34)	0.97 (1.37)
	Processing speed	3.42 (2.45)	4.36 (2.90)	-0.14 (2.10)	0.83 (1.91)	3.37 (2.31)	4.58 (2.91)	5.19 (2.47)	1.39 (2.33)	4.42 (1.80)
	Total score	8.38 (5.89)	7.12 (5.25)	3.52 (8.24)	6.57 (5.60)	8.05 (7.13)	7.63 (6.32)	8.00 (5.12)	8.09 (5.37)	8.18 (5.30)

See Table 1 and Appendix S2 legends.
